# Constituent-based quasi-linear viscoelasticity: a revised quasi-linear modelling framework to capture nonlinear viscoelasticity in arteries

**DOI:** 10.1007/s10237-023-01711-8

**Published:** 2023-05-02

**Authors:** Alessandro Giudici, Koen W. F. van der Laan, Myrthe M. van der Bruggen, Shaiv Parikh, Eline Berends, Sébastien Foulquier, Tammo Delhaas, Koen D. Reesink, Bart Spronck

**Affiliations:** 1 Department of Biomedical Engineering, CARIM School for Cardiovascular Diseases, Maastricht University, Universiteitssingel 40, Room C5.568, 6229 ER Maastricht, The Netherlands; 2 GROW School for Oncology and Reproduction, Maastricht University, Maastricht, The Netherlands; 3 Department of Internal Medicine, CARIM School for Cardiovascular Diseases, Maastricht University, Maastricht, The Netherlands; 4 Department of Pharmacology and Toxicology, CARIM School for Cardiovascular Diseases, Maastricht University, Maastricht, The Netherlands; 5 Macquarie Medical School, Faculty of Medicine, Health and Human Sciences, Macquarie University, Sydney, Australia

**Keywords:** Arterial viscoelastic modelling, Arterial mechanics, Constituent-based quasi-linear viscoelastic modelling, Elastin, Collagen

## Abstract

Arteries exhibit fully nonlinear viscoelastic behaviours (i.e. both elastically and viscously nonlinear). While elastically nonlinear arterial models are well established, effective mathematical descriptions of nonlinear *visco*elasticity are lacking. Quasi-linear viscoelasticity (QLV) offers a convenient way to mathematically describe viscoelasticity, but its viscous linearity assumption is unsuitable for whole-wall vascular applications. Conversely, application of fully nonlinear viscoelastic models, involving deformation-dependent viscous parameters, to experimental data is impractical and often reduces to identifying specific solutions for each tested loading condition. The present study aims to address this limitation: By applying QLV theory at the wall *constituent* rather than at the *whole-wall* level, the deformation-dependent relative contribution of the constituents allows to capture nonlinear viscoelasticity with a unique set of deformation-independent model parameters. Five murine common carotid arteries were subjected to a protocol of quasi-static and harmonic, pseudo-physiological biaxial loading conditions to characterise their viscoelastic behaviour. The arterial wall was modelled as a constrained mixture of an isotropic elastin matrix and four families of collagen fibres. Constituent-based QLV was implemented by assigning different relaxation functions to collagen- and elastin-borne parts of the wall stress. Nonlinearity in viscoelasticity was assessed via the pressure dependency of the dynamic-to-quasi-static stiffness ratio. The experimentally measured ratio increased with pressure, from 1.03 $\pm$ 0.03 (mean $\pm$ standard deviation) at 80–40 mmHg to 1.58 $\pm$ 0.22 at 160–120 mmHg. Constituent-based QLV captured well this trend by attributing the wall viscosity predominantly to collagen fibres, whose recruitment starts at physiological pressures. In conclusion, constituent-based QLV offers a practical and effective solution to model arterial viscoelasticity.

**Supplementary Information:**

The online version contains supplementary material available at 10.1007/s10237-023-01711-8.

## Introduction

The viscoelastic nature of arteries has long been known (Bergel 1961; Dobrin 1978), with *ex vivo* investigations on the mechanical response of the arterial wall to harmonic loading dating as far back as the 1930s (Ranke 1934). Despite these observations, the majority of experimental *ex vivo* studies on arterial wall mechanics have investigated the arterial response to quasi-static loading after preconditioning, thus considering and modelling the arterial wall tissue as a pseudo-elastic material (Giudici et al. 2021c). This choice was likely dictated by two main considerations: (1) the experimental challenges of performing dynamic experiments (van der Bruggen et al. 2021a) and (2) the difficulty of developing computational models that effectively capture the complex viscoelastic response of soft biological tissues (Fung 1997; Amabili et al. 2019a; Franchini et al. 2021). *In vivo*, however, arteries are subjected to dynamic loads (at least in the circumferential direction, if not both axially and circumferentially) (Parikh et al. 2021). Therefore, their *physiological* function differs considerably from that observed in most *experimental* investigations. Indeed, upon dynamic loading, the arterial wall stiffens up to twofold compared to its quasi-static response (Amabili et al. 2019b, 2021; van der Bruggen et al. 2021a; Franchini et al. 2021, 2022b). Moreover, arteries may exhibit pressure-diameter/stress–strain hysteresis (Learoyd and Taylor 1966; Amabili et al. 2019a; van der Bruggen et al. 2021a; Franchini et al. 2021). In *ex vivo* experimental studies in which cyclic loading over wide ranges of deformation is performed, hysteresis is evident (Franchini et al. 2021, 2022b), indicating some degree of energy loss between loading and unloading arms of the pressure-diameter/stress–strain curve. However, under physiological loading conditions (i.e. smaller deformation ranges), the hysteresis area has been shown to be relatively small (Hermeling et al. 2010; Giudici et al. 2021b; van der Bruggen et al. 2021a). Because of these important biomechanical aspects, investigating the viscoelastic mechanical properties of arteries is pivotal to further our understanding of arterial physiology, pathology, and (dys)function.

We previously developed and validated a biaxial testing set-up that allows the characterisation of the mechanical response of arteries under both quasi-static and dynamic loading conditions and using pseudo-physiological loading (i.e. simultaneous pressurisation and axial extension) (van der Bruggen et al. 2021a). This work addressed the experimental considerations. However, given the complexity of the biomechanical data acquired therein, developing mathematical approaches to accurately capture arterial viscoelasticity is the next necessary step for the advancement of the arterial mechanics field. While different mathematical formulations to capture arterial viscoelasticity have been proposed (Armentano et al. 1995b; Fung 1997; Franchini et al. 2021), these approaches are typically either too simple to capture the complex viscoelastic behaviour of arteries, or too complex for a practical application to experimental data.

The simplest approach to viscoelastic modelling is linear viscoelasticity (Fung 1997; Funk et al. 2000). In linear viscoelasticity, it is assumed that the viscoelastic response of a material can be modelled as the superimposed contribution of a finite number of springs and dashpots (i.e. a discrete number of Maxwell, Voigt, or Kelvin models acting in parallel). The simplicity of these models makes them convenient for modelling *in vivo* data on arterial mechanics and function, where the limited available mechanical data complicates informing more complex viscoelastic models. Linear viscoelasticity has been used previously to investigate differences in the viscoelastic behaviour between different locations of the arterial tree in animal models (Armentano et al. 1995b; Bia et al. 2005; Valdez-Jasso et al. 2009), as well as the effect of ageing and hypertension on arterial wall viscoelasticity in humans (Learoyd and Taylor 1966; Armentano et al. 1995a). The linearity of these models, however, constitutes their major limitation (Fung 1997; Funk et al. 2000). Indeed, arteries exhibit highly nonlinear mechanical behaviours (Sommer and Holzapfel 2012; Bellini et al. 2014; Spronck and Humphrey 2019; Giudici et al. 2021c), so that these simple models can only be used to capture arterial mechanics over small pressure/deformation ranges for which the linearity assumption may be reasonable.

To overcome the limitations of linear viscoelasticity, Fung introduced the concept of quasi-linear viscoelasticity (QLV) (Fung 1997). In QLV, the viscoelastic stress at any time instant can be calculated as the convolution integral over the entire deformation history between a reduced relaxation function and the time derivative of the elastic stress. The elastic stress (i.e. the response of the material if it were purely elastic) is defined as a function of the deformation using any, generally nonlinear and anisotropic, strain energy function (SEF) (De Pascalis et al. 2014), thus enabling the model to capture the nonlinear elastic behaviour of soft biological tissues (Gasser et al. 2006; Bellini et al. 2014). The fundamental assumption and simplification of QLV is that linearity is preserved in terms of material viscosity (Fung 1997; De Pascalis et al. 2014; Berjamin et al. 2021). As in the classical stress-relaxation test, a relaxation function is a mathematical expression that captures the gradual decrease of the stress in a viscoelastic material over time following the application of a stepwise change in deformation (Zou and Zhang 2009; Faturechi et al. 2019). The QLV assumption implies that the reduced relaxation function is a function of time only and is unaffected by the applied deformation magnitude (Fung 1997; De Pascalis et al. 2014).

QLV has been used previously to capture the viscoelastic behaviour of different soft biological tissues, including ligaments (Funk et al. 2000; Criscenti et al. 2015), ureter (Wang et al. 2022), articular cartilage (Woo et al. 1980), and also the arterial wall (Craiem et al. 2008; Zou and Zhang 2011; Yang et al. 2011; Pursell et al. 2016; Kermani et al. 2017; Faturechi et al. 2019). However, different studies on arterial viscoelasticity suggest that the fundamental assumption of QLV may fall short in accurately capturing the complex viscoelastic response of the vessel wall. In particular, it has been reported that the stress relaxation of arteries subjected to uniaxial deformation is highly dependent on the magnitude and direction (i.e. circumferential or axial) of the applied deformation (Craiem et al. 2008; Zou and Zhang 2011; Yang et al. 2011). Nonlinear viscoelastic behaviour was also identified by studying the dynamic-to-quasi-static stiffness ratio as well as the hysteresis (van der Bruggen et al. 2021a; Franchini et al. 2021), both of which were found to be strongly dependent on the initial deformation state. For these reasons, fully nonlinear viscoelastic models have been proposed, which involve viscous parameters that are a continuous function of strain (Provenzano et al. 2002; Yang et al. 2011; Franchini et al. 2021). The accurate identification of these continuous functions, however, is not trivial and requires acquiring mechanical data over a broad range of loading conditions for each tested sample. As this is impractical from an experimental standpoint, these approaches are often reduced to identifying different sets of parameter values to describe the response to harmonic loading at different initial deformations and load directions (Provenzano et al. 2002; Yang et al. 2011; Franchini et al. 2021). Hence, while proving useful to analyse the measured mechanical data, these approaches do not allow to predict the wall behaviour for loading conditions which have not been tested.

An important limitation of the aforementioned viscoelastic models consists in the attempt to describe the viscoelastic properties of the arterial wall tissue as a whole, neglecting its complex microstructural composition which is likely at the root of the observed viscoelastic nonlinearities. Constituent-based viscoelasticity addresses this limitation (Holzapfel et al. 2002; Vena et al. 2006; Thomas et al. 2009; Peña et al. 2011). It is based on two key concepts: (1) the viscoelastic behaviour of soft tissues is determined by their microstructural composition, and (2) the assumption of linearity in viscous behaviour is inaccurate at a *whole-tissue* level but may be reasonable at level of the individual tissue *constituents* (e.g. collagen, elastin, and glycosaminoglycans). As the constituents’ relative contributions to the mechanical behaviour of the tissue are deformation-dependent, their different viscoelastic properties weight differently at different deformation levels. Therefore, the resulting viscoelastic behaviour is fully nonlinear and has the advantage of relying on a unique set of constituent-specific, deformation-independent model parameters. Interestingly, recent experimental findings seem to support the potential validity of constituent-based viscoelasticity. Indeed, Zou and Zhang (2011) reported that the load/strain dependency of the stress relaxation of isolated aortic elastin was much reduced compared to that of the intact aortic wall. Furthermore, Amabili et al. (2019a) and Franchini et al. (2021) found differences in viscoelasticity, as assessed by the dynamic-to-quasi-static stiffening ratio and loss factor, between the isolated human aortic intima, media, and adventitia, each, in turn, different from that of the intact wall. They postulated that microstructural and compositional differences between layers likely were at the basis of the differences in viscoelastic behaviours, suggesting that the viscoelastic behaviour of individual wall constituents may be key to understand viscoelasticity at the intact tissue level. Nevertheless, while constituent-based viscoelastic models of the arterial wall have been proposed previously in theoretical studies (Holzapfel et al. 2002; Nedjar 2007; Peña et al. 2011), their application to model experimental data has so far been very limited (Peña et al. 2011).

In the present study, we aimed to develop a constitutive modelling framework that, by combining Fung’s QLV theory with that of constituent-based viscoelasticity, allows to (1) capture the complex nonlinear viscoelastic behaviour of the arterial wall using a unique set of deformation-independent model parameters, and (2) relate the viscoelastic mechanical properties at a whole-tissue level to those of its microstructural constituents. The performance of the proposed constituent-based QLV (cbQLV) model was evaluated by modelling the viscoelastic response of the mouse common carotid artery, also comparing it to that of the standard QLV (sQLV) approach.

## Methods

### Kinematics

**Fig. 1:**
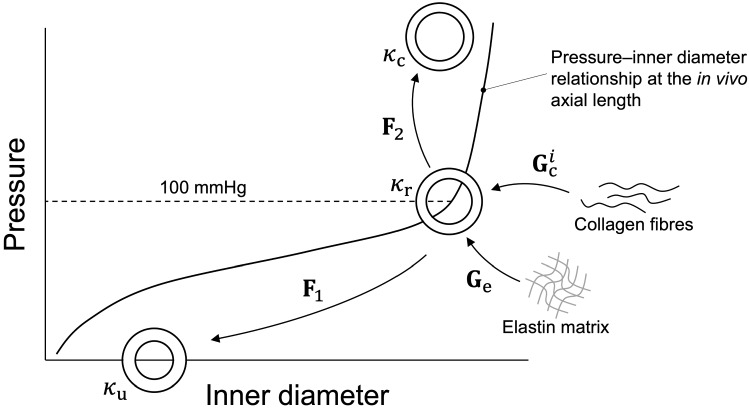
Schematic representation of the configurations and deformation gradients used in this study. Rather than defining deformations with respect to the unloaded configuration (${\kappa }_{\mathrm{u}}$), the reference configuration (${\kappa }_{\mathrm{r}}$) was set to the vessel configuration at the mean physiological pressure of 100 mmHg and at the *in vivo* axial length (${l}_{\mathrm{iv}}$). ${\kappa }_{\mathrm{c}}$ defines a generic non-homeostatic configuration in which luminal pressure, axial length, or both are not 100 mmHg and ${l}_{iv}$, respectively. The deformation gradients **F**_1_ and **F**_2_ map the deformation from ${\kappa }_{\mathrm{r}}$ to ${\kappa }_{\mathrm{u}}$ and ${\kappa }_{\mathrm{c}}$, respectively. The deposition stretch matrices ${\mathbf{G}}_{\mathrm{e}}$ and ${\mathbf{G}}_{\mathrm{c}}^{i}$ map the deformation of the elastin matrix and the collagen fibre family *i*, respectively, from their stress-free configuration to ${\kappa}_{\mathrm{r}}$

The arterial wall was modelled as an incompressible, thin, cylindrical membrane. As proposed previously (Bellini et al. 2014), we chose to define the deformed state during the *ex vivo* experiments with respect to an *in vivo*, homeostatic reference configuration (${\kappa }_{\mathrm{r}}$) rather than the unloaded configuration (${\kappa }_{\mathrm{u}}$, Fig. 1). We then used constituent-specific deposition stretches to define the pre-deformed state of the wall constituents in ${\kappa }_{\mathrm{r}}$. As wall constituents (i.e. elastin and collagen) develop and remodel in the *in vivo* state, this choice allows to define constitutive model parameters that more closely reflect the constituents’ behaviour *in vivo*. We define ${\kappa }_{\mathrm{r}}$ as the configuration of the vessel at its *in vivo* axial length (${l}_{iv}$) and at a physiological mean arterial pressure of 100 mmHg, using cylindrical coordinates $(\rho ,\vartheta ,\mathcal{Z})$ to denote the position of a point in this configuration. It is then useful to introduce the deformation gradient ${\mathbf{F}}_{1}$, mapping the deformation from ${\kappa }_{\mathrm{r}}$ to ${\kappa }_{\mathrm{u}}$ (Fig. 1):

1 $$\begin{array}{c}{\mathbf{F}}_{1}=\text{diag}\left[\frac{\partial R}{\partial \rho },\frac{R\partial\Theta }{\rho \partial \vartheta },\frac{\partial Z}{\partial \mathcal{Z}}\right]=\text{diag}\left[{\Lambda }_{R},{\Lambda }_{\Theta },{\Lambda }_{Z}\right] ,\end{array}$$

where we have used a cylindrical reference system $\left(R,\Theta ,Z\right)$ for ${\kappa }_{\mathrm{u}}$. Note that, given the thin-wall assumption, all deformations refer to the mid-wall coordinate. Furthermore, we assumed deformations to be orthotropic (i.e. no twist occurred in our experiments). Note, also, that because constituent-specific deposition stretches are defined in ${\kappa }_{\mathrm{r}}$, ${\kappa }_{\mathrm{u}}$ is an unloaded but not a stress-free configuration, i.e. individual constituents may be subjected to residual stresses, the summed contributions of which yield null stress in all three principal directions. The axial component of ${\mathbf{F}}_{1}$ is defined as the ratio between the unloaded length of the artery (${L}_{0}$) and ${l}_{iv}$: ${\Lambda }_{Z}={L}_{0}/{l}_{iv}$. The circumferential and radial components of ${\mathbf{F}}_{1}$ are then derived from the incompressibility constraint:

2 $$\begin{array}{c}{\Lambda }_{\Theta }=\frac{{R}_{\mathrm{m}}}{{\rho }_{\mathrm{m}}}=\sqrt{\frac{{R}_{\mathrm{m}}^{2}}{{\rho }_{\mathrm{o}}^{2}-\left({R}_{\mathrm{o}}^{2}-{R}_{\mathrm{m}}^{2}\right){\Lambda }_{Z}}}\end{array}$$

and ${\Lambda }_{R}=1/{\Lambda }_{Z}{\Lambda }_{\Theta }$ (incompressibility). ${R}_{\mathrm{m}}=\left({R}_{\mathrm{i}}+{R}_{\mathrm{o}}\right)/2$, ${R}_{\mathrm{i}}$, and ${R}_{\mathrm{o}}$ are the mid-wall, inner, and outer radii in ${\kappa }_{\mathrm{u}}$; ${\rho }_{\mathrm{m}}$ and ${\rho }_{\mathrm{o}}$ are the mid-wall and outer radii in ${\kappa }_{\mathrm{r}}$. We can then introduce a generic loaded configuration ${\kappa }_{\mathrm{c}}$ in cylindrical coordinates $(r,\theta ,z)$ and define the deformation gradient ${\mathbf{F}}_{2}$ mapping the deformation from ${\kappa }_{\mathrm{r}}$ to ${\kappa }_{\mathrm{c}}$ (Fig. 1):

3 $$\begin{array}{c}{\mathbf{F}}_{2}=\text{diag}\left[\frac{\partial r}{\partial\uprho },\frac{r\partial\uptheta }{\rho \partial \vartheta },\frac{\partial z}{\partial \mathcal{Z}}\right]=\text{diag}\left[{\lambda }_{r},{\lambda }_{\uptheta },{\lambda }_{z}\right].\end{array}$$

Once more, the axial component (${\lambda }_{z}$) of ${\mathbf{F}}_{2}$ can be calculated as the ratio between the vessel length in ${\kappa }_{\mathrm{c}}$ and ${l}_{iv}$, while ${\lambda }_{\theta }$ and ${\lambda }_{r}$ can be derived from incompressibility:

4 $$\begin{array}{c}{\lambda }_{\theta }=\frac{r{}_{\mathrm{m}}}{{\rho }_{\mathrm{m}}}=\sqrt{\frac{{r}_{\mathrm{o}}^{2}-\frac{\left({R}_{\mathrm{o}}^{2}-{R}_{\mathrm{m}}^{2}\right){\Lambda }_{Z}}{{\lambda }_{z}}}{{\rho }_{\mathrm{o}}^{2}-\left({R}_{\mathrm{o}}^{2}-{R}_{\mathrm{m}}^{2}\right){\Lambda }_{Z}}}\end{array}$$

and ${\lambda }_{r}=1/{\lambda }_{z}{\lambda }_{\theta }$, where ${r}_{\mathrm{o}}$ and ${r}_{\mathrm{m}}$ are the outer and mid-wall radii in ${\kappa }_{\mathrm{c}}.$

### Viscoelastic modelling framework

Our viscoelastic modelling framework is based on Fung’s QLV theory (Fung 1997). Given that in QLV a material’s viscoelastic behaviour is independent of the initial deformation, we can introduce a reduced relaxation function $Q(t)$ that is a function of time only and that defines the relaxation of the elastic stress following the application of a stepwise change in deformation; note that $Q\left(0\right)=1$. Given any deformation history that maps the transition from ${\kappa }_{\mathrm{r}}$ to ${\kappa }_{\mathrm{c}}$, the 2nd Piola–Kirchhoff (PK) stress ($\mathbf{S}$) (i.e. the stress defined in the reference configuration ${\kappa }_{\mathrm{r}}$) at any time $t$ can be calculated as

5 $${\mathbf{S}}\left( t \right) = {\mathbf{S}}\left( 0 \right) + \int\limits_{0}^{t} {Q\left( {t - s} \right)\frac{{{\text{d}}{\mathbf{S}}^{{\text{e}}} \left( s \right)}}{{{\text{d}}s}}{\text{d}}s} ,$$

where ${\mathbf{S}}^{\mathrm{e}}$ denotes the 2nd PK elastic stress and $\mathbf{S}\left(0\right)$ is the viscoelastic stress at beginning of the deformation history. Note that $\mathbf{S}\left(0\right)$ is in general nonzero and corresponds to the 2nd PK stress at the beginning of each experiment. We do, however, assume that the time interval between experiments was long enough to guarantee a complete viscous stress relaxation at the beginning of each experiment, so that $\mathbf{S}\left(0\right)$ reflects the complete relaxation of ${\mathbf{S}}^{\mathrm{e}}$ at the stretch level corresponding to the beginning of the experiment (i.e. plateau phase in Supplementary information, Figure S1).

The elastic response of the arterial wall was modelled using a four fibre family strain energy density function ($\Psi )$, accounting for the summed contribution of an isotropic matrix (mainly representing the elastin network) (${\Psi }_{\mathrm{e}}$) and four families of fibres (mainly representing the mechanical behaviour of collagen fibres) (${\Psi }_{\mathrm{c}}$) (Gleason et al. 2008).

6 $$\begin{array}{*{20}c} {\Psi = \Psi _{{\text{e}}} + \Psi _{{\text{c}}} = \frac{\mu }{2}\left( {I_{{1,{\text{e}}}} - 3} \right)^{{1 + \beta }} + \sum\limits_{{i = 1}}^{4} {\frac{{k_{1}^{i} }}{{4k_{2}^{i} }}\left[ {e^{{k_{2}^{i} \left( {I_{{4,{\text{c}}}}^{i} - 1} \right)^{2} }} - 1} \right],} } \\ \end{array}$$

where $\mu$ is an isotropic stiffness-like parameter, ${k}_{1}^{i}$ and ${k}_{2}^{i}$ are a stiffness-like and a nonlinearity parameter for the *i*th fibre family, and $\beta$ controls the functional form of the isotropic matrix strain energy density function. Previous works have modelled elastin’s contribution as either that of a Neo-Hookean material (i.e. $\beta =0$) (Holzapfel et al. 2000; Bellini et al. 2014) or using $\beta =0.50$ (Zulliger et al. 2004a, b). However, it has been shown that the mechanical response of the arterial elastin matrix falls in between these two modelled behaviours (Watton et al. 2009). Based on these findings, we chose to set $\beta =0.15$, leading to a nearly Neo-Hookean behaviour for the isotropic matrix. ${I}_{1,\mathrm{e}}$ is the first invariant of elastin’s right Cauchy-Green tensor ${\mathbf{C}}_{\mathrm{e}}={\mathbf{F}}^{\mathrm{T}}{\mathbf{G}}_{\mathrm{e}}^{\mathrm{T}}{\mathbf{G}}_{\mathrm{e}}{\mathbf{F}}$ (i.e. ${I}_{1,\mathrm{e}}=\mathrm{tr}({\mathbf{C}}_{\mathrm{e}})$), where ${\mathbf{G}}_{\mathrm{e}}$ is the deposition stretch matrix of elastin, defined as

7 $$\begin{array}{c}{\mathbf{G}}_{\mathrm{e}}=\text{diag}\left[\frac{1}{{\lambda }_{\uptheta ,\mathrm{e}}{\lambda }_{z,\mathrm{e}}},{ \lambda }_{\uptheta ,\mathrm{e}},{ \lambda }_{z,\mathrm{e}}\right].\end{array}$$

${\lambda }_{z,\mathrm{e}}$ and ${\lambda }_{\uptheta ,\mathrm{e}}$ denote the axial and circumferential deposition stretches of elastin and define its pre-deformed state in ${\kappa }_{\mathrm{r}}$. Hence, ${\mathbf{G}}_{\mathrm{e}}$ maps the deformation of the elastin matrix from its stress-free configuration to ${\kappa }_{\mathrm{r}}$ (Fig. 1). $I_{{4,{\text{c}}}}^{i} = \left[ {({\mathbf{F}}^{{\text{T}}} {\mathbf{G}}_{{\text{c}}}^{{i}{\text{T}}} {\mathbf{G}}_{{\text{c}}}^{i} {\mathbf{F}} ):\left( {{\mathbf{M}}^{i} \otimes {\mathbf{M}}^{i} } \right)} \right]$ is the fourth invariant of the right Cauchy-Green tensor of fibre family *i*, where ${\mathbf{M}}^{i}=[0,\mathrm{sin}{\alpha }^{i},\mathrm{cos}{\alpha }^{i}$] is the orientation vector of fibre family *i,* with ${\alpha }^{1}=0^\circ$ for axially oriented fibres $, {\alpha }^{2}=90^\circ$ for circumferentially oriented fibres, and ${\alpha }^{\mathrm{3,4}}=\pm \alpha$ for diagonally (*n* = 2) oriented fibres, and ${\mathbf{G}}_{\mathrm{c}}^{i}$ is the deposition stretch matrix for fibre family *i*:

8 $$\begin{array}{c}{\mathbf{G}}_{\mathrm{c}}^{i}=\text{diag}\left[\frac{1}{{\left({\lambda }_{\mathrm{c}}\right)}^{2}\mathrm{cos}{\alpha }^{i}\mathrm{sin}{\alpha }^{i}},{\lambda }_{\mathrm{c}}\mathrm{sin}{\alpha }^{i},{\lambda }_{\mathrm{c}}\mathrm{cos}{\alpha }^{i}\right],\end{array}$$

where ${\lambda }_{\mathrm{c}}$ denotes fibre’s deposition stretch in the fibre direction, assumed to be equal for all fibre families. Similar to ${\mathbf{G}}_{\mathrm{e}}$, ${\mathbf{G}}_{\mathrm{c}}^{i}$ maps the deformation of the fibre family *i* from its stress-free configuration to ${\kappa }_{\mathrm{r}}$ (Fig. 1). Note that, to guarantee the absence of torsion in response to orthotropic deformations/loads, the model parameters pertaining to the two diagonal fibre families must necessarily be equal (i.e. ${k}_{1}^{3}={k}_{1}^{4}$, ${k}_{2}^{3}={k}_{2}^{4}$, and ${\lambda }_{\mathrm{c}}^{3}={\lambda }_{\mathrm{c}}^{4}$) given their symmetric orientation. Additionally, for all fibre families, the nonlinearity parameter ${k}_{2}^{\mathrm{c}}$ replaces ${k}_{2}^{i}$ in Eq. (6) whenever ${I}_{4,\mathrm{c}}^{i}<1$. This allows to differentiate compressive from tensile behaviours of the fibres while maintaining continuity in the slope of the modelled stress-stretch relationships. ${\mathbf{S}}^{\mathrm{e}}$ can be calculated as

9 $$\begin{array}{c}{\mathbf{S}}^{\mathrm{e}}=-p{\mathbf{C}}^{-1}+2\frac{\partial\Psi }{\partial {\mathbf{C}}} ,\end{array}$$

where $p$ is a Lagrange multiplier enforcing incompressibility and ${\mathbf{C}}={\mathbf{F}}^{\mathrm{T}}{\mathbf{F}}$.

A relevant viscoelastic property of many biological soft tissues is that the hysteresis behaviour is relatively insensitive to the loading frequency (Fung 1997; Franchini et al. 2021). Commonly used viscoelastic models, such as the Kelvin model, are formulated using a single dashpot in combination with 1 or 2 springs. As a result, the obtained relaxation function follows an exponential decay governed by a single time constant which leads to the hysteresis behaviour being highly dependent on the loading frequency (Fung 1997). This limitation can be overcome by superimposing a large number of simple viscoelastic models to achieve a continuous relaxation spectrum (i.e. structural damping). In this context, Fung proposed the following reduced relaxation function (Fung 1997):

10 $$Q\left( t \right) = \left[ {1 + \nu \left( { \mathop \int \limits_{{\frac{t}{{\tau _{2} }}}}^{{ + \infty }} {\frac{{{\text{e}}^{{ - m}} }}{m}{\text{d}}m - \mathop \int \limits_{{\frac{t}{{\tau _{1} }}}}^{{ + \infty }} {\frac{{{\text{e}}^{{ - m}} }}{m}{\text{d}}m} } } \right)} \right]\left[ {1 + \nu {\text{ln}}\left( {\frac{{\tau _{2} }}{{\tau _{1} }}} \right)} \right]^{{ - 1}} ,$$

where $\nu$ is a dimensionless parameter and ${\tau }_{1}$ and ${\tau }_{2}$ are time constants (${\tau }_{2}>{\tau }_{1}$). $Q\left(t\right)$ yields a relatively constant damping for loading frequencies in the range $1/{\tau }_{2}$–$1/{\tau }_{1}$ (Fung 1997). In the sQLV theory, $Q\left(t\right)$ is used to describe the stress relaxation of the soft biological tissue as a whole. Since $G$ is independent from $\mathbf{C}$, this formulation assumes that the tissue’s viscoelastic behaviour is independent from its deformation state. In the present paper, we expand on Fung’s QLV theory by applying QLV at a *constituent* rather than at a *whole-tissue* level. As in the sQLV formulation, we assume that the viscous response of elastin and collagen fibres is independent from their deformation state but, unlike in sQLV, we define individual relaxation functions ${Q}_{\mathrm{e}}(t)$ and ${Q}_{\mathrm{c}}(t)$ for elastin and collagen, so that their viscous behaviours may differ. Using Eq. (6) and the cbQLV principle, we can then reformulate Eq. (5) to express the Cauchy stress (i.e. the stress defined in the current configuration ${\kappa }_{\mathrm{c}}$) as

11 $$\begin{array}{*{20}c} \begin{gathered} \sigma_{ii} \left( t \right) = \left\{ {S_{ii} \left( 0 \right) + \mathop \int \limits_{0}^{t} \left[ {Q_{{\text{e}}} \left( {t - s} \right)\frac{{\text{d}}}{{{\text{d}}s}}\left( { - \frac{{p_{{\text{e}}} }}{{\lambda_{i}^{2} \left( s \right)}} + 2\frac{{\partial {\Psi }_{{\text{e}}} \left( s \right)}}{{\partial \lambda_{i}^{2} \left( s \right)}}} \right)} \right.} \right. \hfill \\ \quad \quad \quad \quad + \left. {\left. {Q_{{\text{c}}} \left( {t - s} \right)\frac{{\text{d}}}{{{\text{d}}s}}\left( { - \frac{{p_{{\text{c}}} }}{{\lambda_{i}^{2} \left( s \right)}} + 2\frac{{\partial {\Psi }_{{\text{c}}} \left( s \right)}}{{\partial \lambda_{i}^{2} \left( s \right)}}} \right)} \right]{\text{d}}s} \right\}\lambda_{i}^{2} \left( t \right) \, , \hfill \\ \end{gathered} \\ \end{array}$$

with $i\in \left\{r,z,\uptheta \right\}$ (no sum on *i*) and ${p}_{\mathrm{e}}$ and ${p}_{\mathrm{c}}$ Lagrange multipliers for the elastin- and collagen-borne parts of the wall stress. Assuming a complete relaxation of the previous stress history at $t=0$, ${S}_{ii}\left(0\right)$ can be calculated from ${Q}_{\mathrm{e}}\left(t\to \infty \right)$ and ${Q}_{\mathrm{c}}\left(t\to \infty \right)$:

12 $$\begin{array}{c}{S}_{ii}\left(0\right)=\left(-\frac{{p}_{\mathrm{e}}}{{\lambda }_{i}^{2}\left(0\right)}+2\frac{\partial {\Psi }_{\mathrm{e}}\left(0\right)}{\partial {\lambda }_{i}^{2}\left(0\right)}\right){\left[1+{\nu }_{\mathrm{e}}\mathrm{ln}\left(\frac{{\tau }_{2,\mathrm{e}}}{{\tau }_{1,\mathrm{e}}}\right)\right]}^{-1}+\left(-\frac{{p}_{\mathrm{c}}}{{\lambda }_{i}^{2}\left(0\right)}+2\frac{\partial {\Psi }_{\mathrm{c}}\left(0\right)}{\partial {\lambda }_{i}^{2}\left(0\right)}\right){\left[1+{\nu }_{\mathrm{c}}\mathrm{ln}\left(\frac{{\tau }_{2,\mathrm{c}}}{{\tau }_{1,\mathrm{c}}}\right)\right]}^{-1}, \end{array}$$

where ${\nu }_{j}$, ${\tau }_{1,j}$, and ${\tau }_{2,j}$ with $j=\{\mathrm{e},\mathrm{c}\}$ indicate the parameters of ${Q}_{\mathrm{e}}$ and ${Q}_{\mathrm{c}}$ (Eq. 10). Because the relative contributions of elastin and collagen to the total elastic wall stress are deformation-dependent (i.e. dependent on the functional form and parameter values of ${\Psi }_{\mathrm{e}}$ and ${\Psi }_{\mathrm{c}}$), the effects of the two relaxation functions ${Q}_{\mathrm{e}}$ and ${Q}_{\mathrm{c}}$ on the total viscoelastic stress are differentially weighted at different deformations. For example, for deformation levels at which elastin bears most of the load, the wall will exhibit a viscoelastic response which is mostly dictated by the function ${Q}_{\mathrm{e}}$. The opposite holds for collagen-dominated deformation levels. Therefore, while retaining the quasi-linear formulation of sQLV, the proposed formulation allows to capture more complex fully nonlinear viscoelastic behaviours.

### Experimental data

The proposed viscoelastic modelling framework was used to capture the mechanical behaviour of the mouse carotid artery. Five surplus male C57BL/6 J-*Glo1* wild type mice were euthanized at an age of 18 weeks using an overdose of isoflurane followed by exsanguination. Thereafter, their left common carotid artery was harvested for mechanical testing. The use of surplus animals, after euthanasia, has been approved by the Maastricht University Animal Ethical Committee (licence number: AVD1070020187086) and all experiments were performed in accordance with the European Union Directives for animal experiments. The biaxial testing set-up used herein is equivalent to that described and characterised in a previous study (van der Bruggen et al. 2021a), except for the method used for the measurement of the diameter distension, here performed via a high speed camera (frame rate 500 Hz) rather than ultrasound scanning. Briefly, our biaxial set-up allows to simultaneously subject tubular vessels to prescribed axial stretches and intraluminal pressures while continuously recording the outer diameter via video tracking and the axial force via a load cell. The sampling frequency of the data acquisition (DAQ) system used to record all variables other than diameter was 2 kHz. After preconditioning (see Supplemental information), the vessel’s *in vivo* axial length (${l}_{iv,\mathrm{exp}}$) was experimentally estimated as that length that guarantees decoupling between axial force and intraluminal pressure (Van Loon et al. 1977; van der Bruggen et al. 2021a). The testing protocol involved three blocks of experimental steps. First, the vessel was subjected to three quasi-static inflation/deflation pressure sweeps. In these three experimental steps, the intraluminal pressure was slowly increased and then decreased (inflation rate ~ 3 mmHg/s) between 10 and 180 mmHg, while keeping the artery at a constant axial length of 105%, 95%, and 100% of ${l}_{iv,\mathrm{exp}}$, respectively. Second, while keeping the vessel axially stretched to ${l}_{iv,\mathrm{exp}}$, the vessel was subjected to trains of sinusoidally oscillating pressure at four different frequencies ($f=2.5, 5, 10,$ and 20 Hz) and at three different pressure ranges: 80–40 mmHg (low), 120–80 mmHg (medium), and 160–80 mmHg (high). Hence, the second testing block yields a total of $4\times 3=12 f$–pressure combinations. Note that 10 Hz = 600 beats per minute is commonly assumed to be the physiological heart rate of mice (Leloup et al. 2016; Ferruzzi et al. 2018). Third, the vessel was subjected to five quasi-static axial force sweeps (stretch rate = 0.11 s^−1^) between ${F}_{z}=0$ g and the maximum axial force reached during the quasi-static pressure sweep at $l=1.05$ ${l}_{iv,\mathrm{exp}}$ and while maintaining a constant intraluminal pressure of 10, 60, 100, 140, or 180 mmHg, respectively. The complete protocols, thus, include 20 different biaxial testing datasets (8 quasi-static and 12 dynamic).

#### Signal alignment

Correct phase and time alignment of the measured signals is of importance when assessing the viscoelastic behaviour of a material (van der Bruggen et al. 2021a). While the experimental set-up used herein has been developed to minimise instrumentation-induced phase shifts between the acquired pressure and diameter signals (van der Bruggen et al. 2021a), the high loading frequencies that are necessary to assess pseudo-physiological arterial function in mice (i.e. heart rate ~ 10 Hz) imply that even small delays will significantly affect the relationships between signals. In particular, two possible sources of delay are worth considering. First, the camera and the DAQ system operate independently. A rectangular 1 ms-long trigger pulse was generated by the camera when acquiring each frame, which was recorded at 2 kHz by the DAQ for synchronisation of the two systems. In the worst-case scenario, this could lead to an inter-system phase difference of up to 0.5 ms (i.e. the sampling interval of the DAQ). Second, the intraluminal pressure was measured via a sensor placed downstream to the distal pipette to which the artery was tethered (van der Bruggen et al. 2021a). Conversely, the diameter waveform was tracked approximately in the centre of the vessel (i.e. nearly at the same distance between proximal and distal pipettes). As the pressure wave propagates through the vessel, this configuration leads to a delay between the acquired pressure and diameter waveforms that can be estimated as the ratio between half of the vessel axial length and the propagation speed of the pressure waveform along the vessel. Although this delay is intrinsically axial length- and pressure-dependent, for an average wave speed of 5 m/s and loaded axial length of 10 mm, the delay is on the order of 1 ms. It is apparent that the effect of these delays on the signal alignment becomes more relevant as the loading frequency increases. Therefore, to correct for these delays, we assumed their effect to be negligible at the lowest harmonic loading frequency included in our protocol (2.5 Hz). Then, based on the empirical observations that loss factor (refer to Section 2.5 and Supplementary information, Figure S2) of soft biological tissues is nearly independent of the loading rate (Fung 1997; Franchini et al. 2021), we adjusted the signal alignment to attain this behaviour. In agreement with the analysis above, the delays needed for the adjustment were $\le$ 2 ms.

### Parameter estimation

The proposed cbQLV constitutive model comprises a total of eighteen model parameters. Twelve of these belong to the elastic part of the model, with ${\Psi }_{\mathrm{e}}$ (Eqs. 6 and 7) including three model parameters (one stiffness-like parameter and two deposition stretches for the circumferential and axial direction, respectively) and ${\Psi }_{\mathrm{c}}$ (Eqs. 6 and 8) involving nine parameters (one stiffness-like parameter and one nonlinearity parameter for each collagen fibre family (the two diagonal families share the same parameters); one orientation angle for diagonally oriented fibre families; one nonlinearity parameter for all compressed fibres; and one deposition stretch in the fibre direction). The remaining six parameters belong to the viscous part of the model, with three model parameters for both ${Q}_{\mathrm{e}}(t)$ and ${Q}_{\mathrm{c}}(t)$ (Eq. 10). Despite the extensive static and dynamic characterisation performed herein, some of these model parameters had to be constrained not to incur into overfitting. Pertaining the elastic part of the model, in a preliminary analysis, we observed that, if left unconstrained, ${\lambda }_{z,\mathrm{e}}$ tended to converge to high values (range 7–10), suggesting that the elastin matrix is subjected to a seven-to-tenfold elongation with respect to its stress-free configuration in ${\kappa }_{\mathrm{r}}$. As these values are non-physiological, we chose to fix ${\lambda }_{z,\mathrm{e}}$ to ${l}_{iv,\mathrm{exp}}/{L}_{0}$; i.e. we assumed elastin not to be axially stretched in ${\kappa }_{\mathrm{u}}$. Further, we chose to set ${k}_{2}^{\mathrm{c}}={10}^{-6}$, thus limiting the collagen’s response to compression by conferring a nearly quadratic, rather than exponential, dependency of the collagen 2nd PK stress on the deformation in compression. Additionally, we constrained the stiffness-like parameter of all fibre families to be equal: ${k}_{1}^{1}={k}_{1}^{2}={k}_{1}^{\mathrm{3,4}}$. Pertaining the viscous part of the model, the two time constants in Eq. (10) define the frequency band within which the material exhibits a stable hysteresis area. Previous studies have shown that the identification of these time constants from stress-relaxation experiments is not trivial (Funk et al. 2000). Alternatively, an accurate estimation of ${\tau }_{1}$ and ${\tau }_{2}$ would require subjecting the vessel to dynamic testing over a very wide range of loading frequencies (~ 10^–4^–10^4^ Hz). This last approach is hardly achievable experimentally, especially for high frequencies. To address this issue, we adopted a more pragmatic approach for the estimation of ${\tau }_{1}$ and ${\tau }_{2}$ which is based on experimental observation of the presence of residual hysteresis effect in quasi-static protocol steps (where the loading rate was nearly 2 orders of magnitude lower than the dynamic protocol steps at the lowest frequency), suggesting that viscoelastic effects are not negligible even in quasi-static loading conditions. Therefore, we chose to fix ${\tau }_{1}={10}^{-3}$ s for both ${Q}_{\mathrm{e}}(t)$ and ${Q}_{\mathrm{c}}(t)$ (i.e. above the inverse of the highest loading frequency used in our protocol, 20 Hz), while fitting ${\tau }_{2}$ to match the level of residual viscoelasticity observed in the quasi-static protocol steps. This guarantees a stable hysteresis behaviour within the loading frequency range $1/{\tau }_{2}$–$1000$ Hz.

**Fig. 2:**
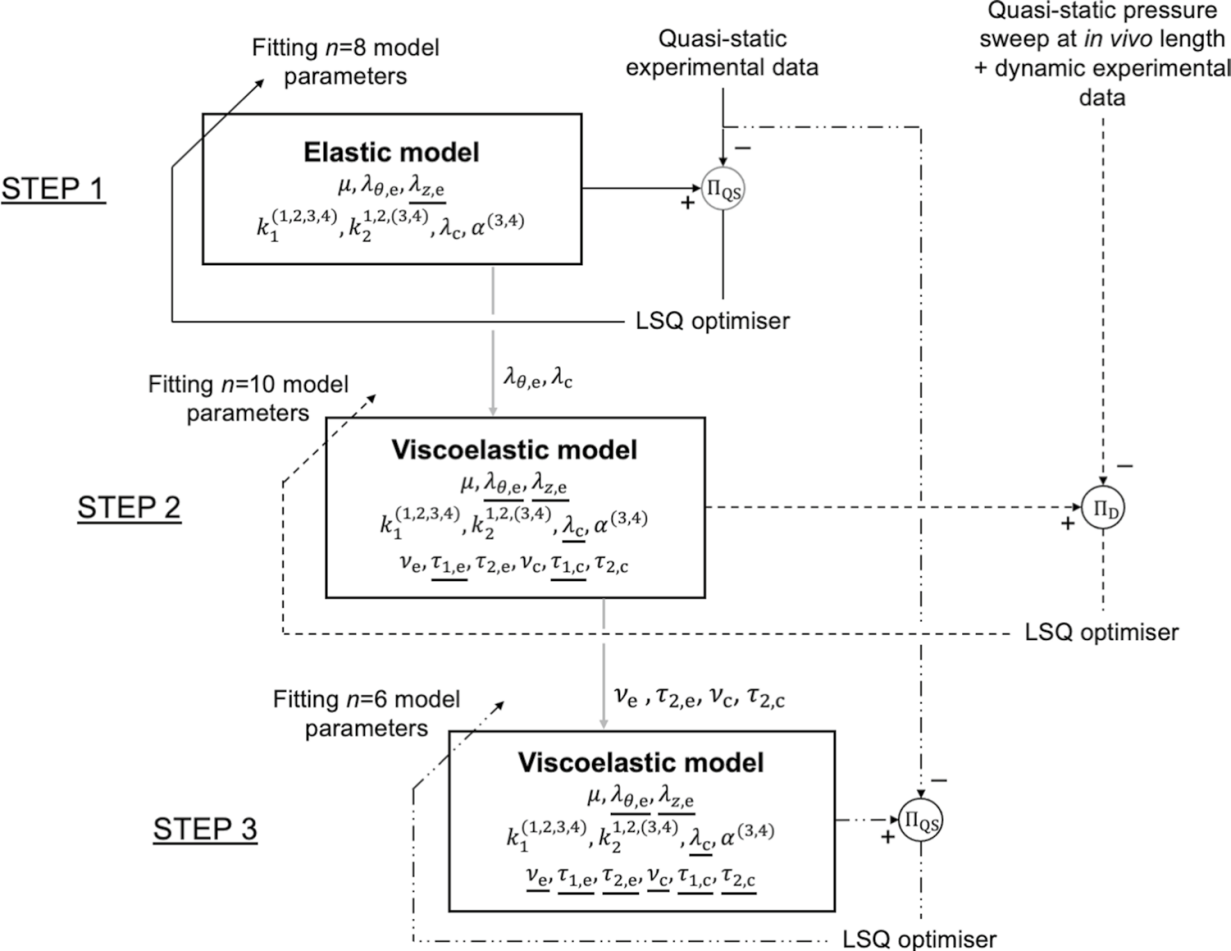
Schematic representation of the three-step parameter fitting procedure implemented to estimate the 18 model parameters of the proposed constituent-based quasi-linear viscoelastic model. Solid, dashed, and dashed-dotted lines are used to indicate the first, second, and third step, respectively. Underlined model parameters indicate fixed parameters. LSQ: least-squares. Brackets in parameter superscripts indicate that the parameter is equal for the enclosed fibre families: e.g. ${k}_{1}^{(\mathrm{1,2},\mathrm{3,4})}$ indicates ${k}_{1}^{1}={k}_{1}^{2}={k}_{1}^{3}={k}_{1}^{4}$

**Fig. 3:**
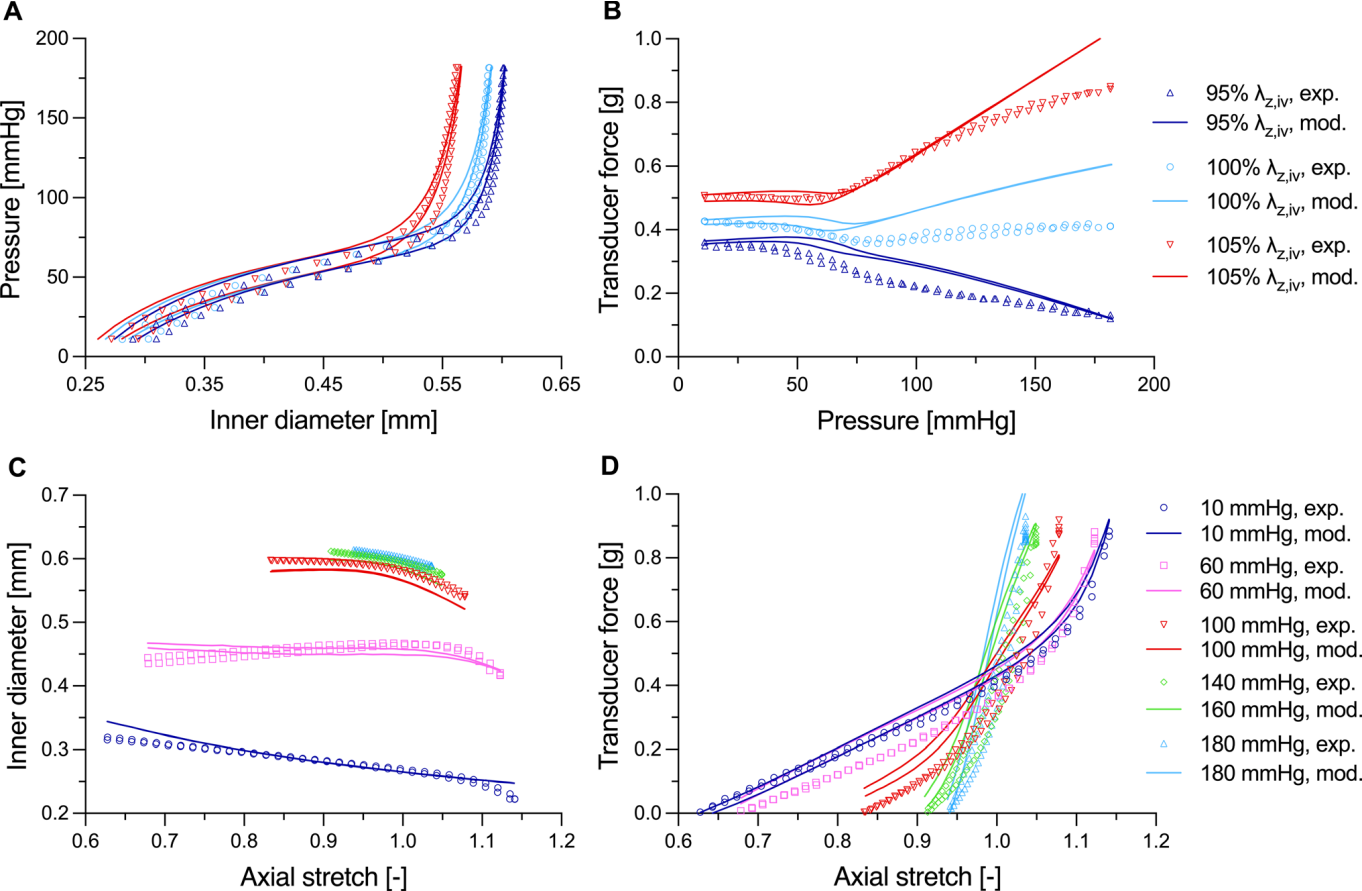
Constituent-based quasi-linear viscoelasticity (cbQLV) captures quasi-static biaxial vessel behaviours well as evidenced by the good agreement between experimental (exp.) and modelled (mod.) curves in Panels **A**–**D**. Panels **A** and **B** show the comparison between experimental and cbQLV-modelled curves of the quasi-static pressure sweeps at constant axial stretch and panels **C** and **D** show axial force sweeps at constant intraluminal pressure of the carotid artery of mouse II. Note that the axial stretch in panels **C** and **D** is expressed with respect to an *in vivo* reference configuration. As a result, axial stretch = 1 is attained at the crossover point between the axial force sweep curves when pressure and axial force are decoupled

The fitting procedure to estimate the remaining twelve model parameters was carried out in three consecutive steps (Fig. 2). First, a purely elastic model (i.e. the eight unconstrained model parameters in $\Psi$: $\mu$, ${\lambda }_{\theta ,\mathrm{e}}$, ${k}_{1}^{\mathrm{1,2},\mathrm{3,4}}$, ${k}_{2}^{1}$, ${k}_{2}^{2}$, ${k}_{2}^{\mathrm{3,4}}$, ${\alpha }^{\mathrm{3,4}}$, and ${\lambda }_{\mathrm{c}}$) was fitted to the quasi-static experimental data (i.e. three pressure sweeps and five axial force sweeps). Loading and unloading curves of each protocol step were resampled (*N* = 35 datapoints for both loading and unloading) and averaged to yield a representative purely elastic behaviour. Then, the model parameters were estimated iteratively by minimising the cost function

13 $$\begin{array}{c}{\Pi }_{\mathrm{QS}}=\sum\limits_{m=1}^{M}{w}_{m}\left[\sum\limits_{n=1}^{N}{\left(\frac{{P}_{\mathrm{comp},n}-{P}_{\mathrm{exp},n}}{{\overline{P} }_{\mathrm{exp}}}\right)}^{2}+\sum\limits_{n=1}^{N}{\left(\frac{{F}_{\mathrm{comp},n}-{F}_{\mathrm{exp},n}}{{\overline{F} }_{\mathrm{exp}}}\right)}^{2}\right] ,\end{array}$$

where ${w}_{m}$ is a weighting factor for protocol step *m* ($M=8$ is the total number of quasi-static protocol steps), ${P}_{\mathrm{comp},n}$ and ${P}_{\mathrm{exp},n}$ are modelled and experimental pressure data points, ${\overline{P} }_{\mathrm{exp}}$ is the average experimental pressure over the entire quasi-static protocol, ${F}_{\mathrm{comp},n}$ and ${F}_{\mathrm{exp},n}$ are modelled and experimental transducer force data points, and ${\overline{F} }_{\mathrm{exp}}$ is the average experimental transducer force over the entire quasi-static protocol. Then, ${w}_{m}$ was set to 1 for each of the three pressure sweeps, while the five axial force sweeps were given a cumulative weight of 1. This differential weighting of axial and pressure sweep protocol steps was used to prioritise steps conducted in pseudo-physiological loading conditions in the search for material parameters. Furthermore, each axial sweep protocol step was attributed a relative weight that was proportional to the explored deformation space in the circumferential-axial plane (i.e. proportional to the length of the circumferential stretch-axial stretch relationship of each protocol step in Fig. 3C). Using Eq. (9), the modelled pressure and transducer force were calculated from thin-wall approximation of equilibrium equations in the radial and axial directions, respectively:

14 $$P_{{{\text{comp}}}} = \int\limits_{{r_{{\text{i}}} }}^{{r_{{\text{o}}} }} {\frac{{\sigma _{{\theta \theta }} - \sigma _{{rr}} }}{r}{\text{d}}r} \approx \left( {\sigma _{{\theta \theta }} - \sigma _{{rr}} } \right)\frac{h}{{r_{{\text{m}}} }},{\text{ and}}$$

15 $$F_{{{\text{comp}}}} = \pi\int\limits_{{r_{{\text{i}}} }}^{{r_{{\text{o}}} }} {\left( {2\sigma _{{zz}} - \sigma _{{\theta \theta }} - \sigma _{{rr}} } \right)r{\text{d}}r} \approx \pi\left( {2\sigma _{{zz}} - \sigma _{{\theta \theta }} - \sigma _{{rr}} } \right)r_{{\text{m}}} h~,$$

where $h={r}_{\mathrm{o}}-{r}_{\mathrm{i}}$ is the wall thickness. This first optimisation step was used to estimate and then fix ${\lambda }_{\mathrm{c}}$ and ${\lambda }_{\theta ,\mathrm{e}}$. As these parameters define the degree of collagen and elastin prestretch in ${\kappa }_{r}$, we assumed that their estimation could be performed independently of the viscoelastic formulation.

The second step of the fitting procedure consisted of fitting the complete viscoelastic model (i.e. the remaining 6 unconstrained parameters in $\Psi$: $\mu$, ${k}_{1}^{\mathrm{1,2},\mathrm{3,4}}$, ${k}_{2}^{1}$, ${k}_{2}^{2}$, ${k}_{2}^{\mathrm{3,4}}$, ${\alpha }^{\mathrm{3,4}}$ given, ${k}_{2}^{\mathrm{c}}={10}^{-6}$, ${\lambda }_{z,\mathrm{e}}={l}_{iv,\mathrm{exp}}/{L}_{0}$, and ${\lambda }_{\vartheta ,\mathrm{e}}$ and ${\lambda }_{\mathrm{c}}$ from fitting step 1) and $\nu$ and ${\tau }_{2}$ for both elastin (${\nu }_{\mathrm{e}}$ and ${\tau }_{2,\mathrm{e}}$) and collagen (${\nu }_{\mathrm{c}}$ and ${\tau }_{2,\mathrm{c}}$) on all protocol steps conducted at ${l}_{iv,\mathrm{exp}}$; i.e. one quasi-static pressure sweep and twelve dynamic loops. The aim of this step was to estimate the viscous parameters that best capture the transition from quasi-static to dynamic behaviour at the *in vivo* axial stretch, especially with respect to the dynamic stiffening. Note that, unlike in the previous step, here, the loading and unloading parts of the quasi-static data are not averaged but are both captured by the viscoelastic model. The model parameters were iteratively estimated by minimising the cost function

16 $$\begin{array}{*{20}c} {\Pi _{{\text{D}}} = \Pi _{{{\text{QS}}}} + \sum\limits_{{j = 1}}^{J} {\left( {\frac{{{\mathcal{K}}_{{{\text{D}},{\text{mod}},j}} }}{{{\mathcal{K}}_{{{\text{QS}},{\text{mod}},j}} }} - \frac{{{\mathcal{K}}_{{{\text{D}},{\text{exp}},j}} }}{{{\mathcal{K}}_{{{\text{QS}},{\text{exp}},j}} }}} \right)^{2} } } \\ \end{array} ,$$

where ${\mathcal{K}}_{\mathrm{D},\mathrm{mod}}$ and ${\mathcal{K}}_{\mathrm{QS},\mathrm{mod}}$ are the dynamic and quasi-static circumferential tangential elastic moduli derived from the modelled relationship for loop $j$, ${\mathcal{K}}_{\mathrm{D},\mathrm{exp}}$ and ${\mathcal{K}}_{\mathrm{QS},\mathrm{exp}}$ are corresponding values calculated from the experimental data, and $J=12$ is the number of dynamic loops. For both experimental and modelled curves, ${\mathcal{K}}_{\mathrm{D}}$ was estimated as the slope of the linear regression of the Cauchy stress–strain dynamic loop, while the corresponding ${\mathcal{K}}_{\mathrm{QS}}$ was determined as the mean of the slopes of the Cauchy stress–strain quasi-static relationship during loading and unloading in the same pressure range as the dynamic loop. In order to illustrate the difference between cbQLV and sQLV, this second step in the fitting procedure was repeated for ${\nu }_{\mathrm{e}}={\nu }_{\mathrm{c}}$.

The third and final step of the fitting procedure aimed to refine the estimation of the six unconstrained elastic model parameters given ${\nu }_{\mathrm{e}}$, ${\tau }_{2,\mathrm{e}}$, ${\nu }_{\mathrm{c}}$, and ${\tau }_{2,\mathrm{c}}$. This was done by fitting the viscoelastic model onto all quasi-static data, iteratively minimising Eq. 13. Once more, unlike in the first optimisation step, loading and unloading parts of the protocol steps were considered independently.

The minimisation task of each of the three steps was repeated for 50 initial guesses of model parameters using the *MultiStart* function of MATLAB R2021b (MathWorks, Inc., Natick, MA, USA).

### Performance of constitutive-based quasi-linear viscoelasticity and its comparison to standard quasi-linear viscoelasticity

After the fitting routine was completed, the viscoelastic model was used to simulate the performed biaxial experiments. The overall quality of the fitting was evaluated using the root mean square error (RMSE), defined as ${\text{RMSE}} = \Pi _{{{\text{QS}}}} /(N \cdot M)$, where $N$ and $M$ indicate the number of datapoints in each protocol step and the number of protocol steps, respectively. Then, to evaluate the ability of the model to capture the viscoelastic behaviour of the arterial wall, the dynamic-to-quasi-static stiffness ratio (${\mathcal{K}}_{\mathrm{D}}/{\mathcal{K}}_{\mathrm{QS}}$) and loss factor, defined as the ratio between loss energy (stress–strain hysteresis area) and stored elastic energy (the average area underneath a quarter of a stress–strain cycle multiplied by $2\pi$, see Supplementary information, Figure S2) (Amabili et al. 2019a; Franchini et al. 2021), were calculated for all the considered loading frequencies and pressure conditions, and both using the experimental and simulated data. The loss factor, thus, provides information on the efficiency of the arterial system, relating the amount of energy that is dissipated to the amount of elastic energy that is stored by the artery in a cardiac cycle.

To illustrate the differences between cbQLV and sQLV, we simulated the arterial viscoelastic response to a sinusoidal pressure waveform of 0.5 mmHg of amplitude, centred at pressures ranging 20–180 mmHg in 5 mmHg increments, and at frequencies ranging from 10^–5^–10^5^ Hz in 0.5 increments on a logarithmic scale. Note that, given the choices made for ${\tau }_{1}$ and ${\tau }_{2}$, a loading frequency of ${10}^{-5}$ Hz (i.e. $\ll 1/{\tau }_{2}$ Hz) ensures that all viscous effects are negligible, so that the resulting behaviour can be considered fully static. Similarly, a loading frequency of ${10}^{5} \mathrm{Hz}$ (i.e. $\gg 1/{\tau }_{1}= {10}^{3}$ Hz) yields the fully elastic response of the material. These simulations were used to characterise the response of the two viscoelastic models by evaluating the dynamic-to-static circumferential stiffness ratio (${E}_{\mathrm{D}}/{E}_{\mathrm{S}}$) in terms of the slope of the 2nd PK stress–Green Lagrange strain relationship, using $f={10}^{-5}\mathrm{ Hz}$ as fully static reference. As the QLV theory is formulated in terms of 2nd PK stress, ${E}_{\mathrm{D}}/{E}_{\mathrm{S}}$ guarantees the best visualisation of the differences between cbQLV and sQLV. The elastin- and collagen-specific contributions to the wall viscosity were evaluated by recalculating ${E}_{\mathrm{D}}/{E}_{\mathrm{S}}$ when setting ${\nu }_{\mathrm{c}}=0$ and ${\nu }_{\mathrm{e}}=0$, respectively. Note that although in sQLV ${{\nu }_{\mathrm{e}}=\nu }_{\mathrm{c}}$, this step was performed also in sQLV to illustrate the individual contribution of collagen and elastin.

## Results

### Constitutive-based quasi-linear viscoelastic model

**Fig. 4:**
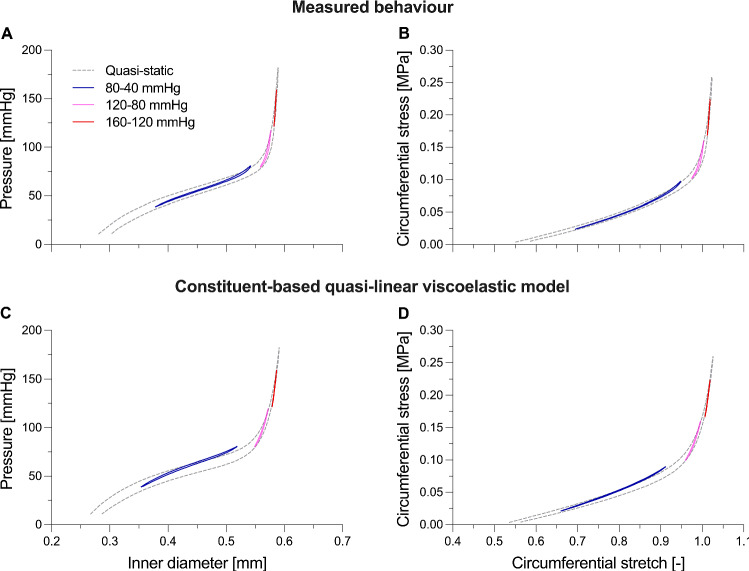
Measured viscosity-related effects are represented by the constituent-based quasi-linear viscoelastic (cbQLV) model, showing comparable patterns between quasi-static and dynamic loading. Dynamic loops at 10 Hz and at the three considered pressure levels (80–40, 120–80, and 160–120 mmHg) of the carotid artery of mouse II are shown, comparing experimental curves (panels **A** and **B**) and those obtained with the cbQLV model (panels **C** and **D**). The quasi-static pressure sweeps at the *in vivo* axial length are also shown as reference to illustrate the difference between quasi-static and dynamic behaviour. Harmonic loading at 10 Hz induces significant stiffening (i.e. increased stress-stretch slope) compared to quasi-static curves in the same pressure range

**Fig. 5:**
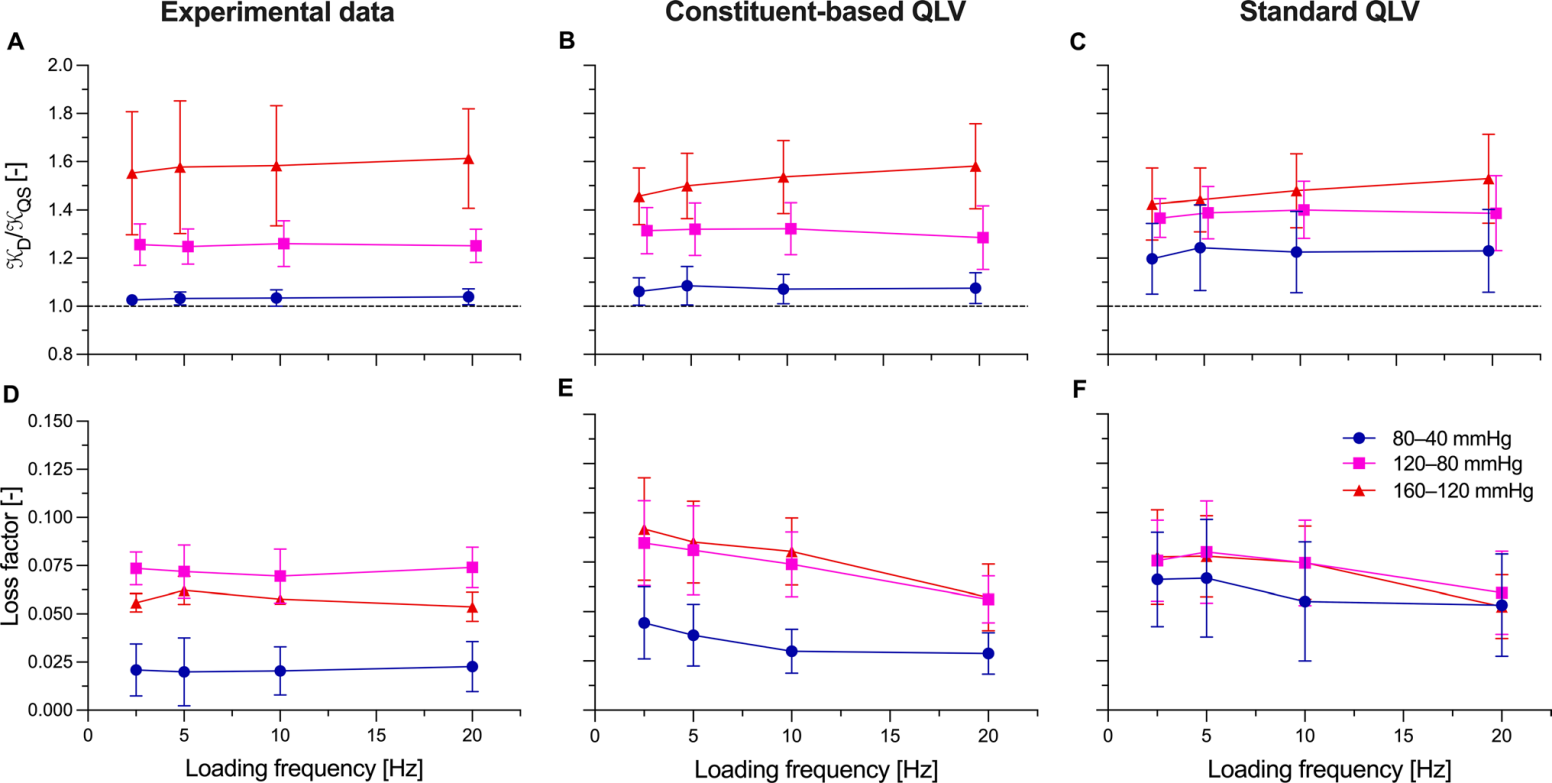
Constituent-based quasi-linear viscoelasticity (cbQLV) effectively captures the pressure dependence of the viscoelastic properties of the mouse carotid artery. Dynamic-to-quasi-static stiffness ratio (Panels **A**–**C**) and loss factor (Panels **D**–**F**) as a function of loading frequency for the experimental data (Panels **A** and **D**), constituent-based quasi-linear viscoelastic model (Panels **B** and **E**) and standard quasi-linear viscoelastic model (Panels **C** and **F**). Data are presented as mean $\pm$ standard deviation

The unloaded outer diameter and wall thickness of the tested mouse carotid arteries were 470 $\pm$ 8 $\upmu$m and 75 $\pm$ 11 $\upmu$m (mean $\pm$ standard deviation), respectively. Figures 3 and 4 show examples of the measured quasi-static and dynamic biaxial mechanical data, as well as corresponding curves simulated with the proposed cbQLV model. The model parameters for all tested arteries are reported in Table 1. While due to the strong nonlinearity of the mechanical behaviour of the mouse carotid arteries the modelled behaviour differed slightly from the measured one in some areas of the biaxial deformation space (e.g. high-pressure range in Fig. 3B), overall, the cbQLV model was able to capture well the complex biaxial response of the mouse carotid artery (Figs. 3 and 4), with an average RMSE of 0.064 $\pm$ 0.020 and 0.066 $\pm$ 0.017 for the dynamic and quasi-static fitting (i.e. steps 2 and 3 in Fig. 2), respectively. As apparent in Fig. 3, despite the inflation rate being ~ 2–3 orders of magnitude slower than that *in vivo*, viscoelastic effects were non-negligible in quasi-static protocol steps, with loading and unloading following clearly distinct paths in all tested arteries. Our cbQLV model attributed the viscous behaviour of the mouse carotid artery predominantly to the fibrous part of $\Psi$ (representing mainly collagen fibres), with four of five arteries having ${\nu }_{\mathrm{e}}=0$ (Table 1). The estimated relaxation of the elastin-borne stress at $t\to +\infty$ was on average 6 $\pm$ 11% (Supplementary information, Figure S1). On the other hand, the fibres’ viscoelastic constant ${\nu }_{\mathrm{c}}$ was 0.099 $\pm$ 0.034, which together with ${\tau }_{2,\mathrm{c}}$ (44.0 $\pm$ 22.5 s), leads to an average stress relaxation of 49 $\pm$ 8% for the fibre-borne part of the stress (Eq. 11). These findings are not surprising when considering differences in ${\mathcal{K}}_{\mathrm{D},\mathrm{exp}}/{\mathcal{K}}_{\mathrm{QS},\mathrm{exp}}$ between the tested pressure ranges (Fig. 5A). In the low-pressure range (80–40 mmHg), where elastin dominates the mechanical behaviour, the arteries underwent marginal stiffening with dynamic loading: ${\mathcal{K}}_{\mathrm{D},\mathrm{exp}}/{\mathcal{K}}_{\mathrm{QS},\mathrm{exp}}$ was 1.03 $\pm$ 0.03 at the physiological heart frequency of 10 Hz. ${\mathcal{K}}_{\mathrm{D},\mathrm{exp}}/{\mathcal{K}}_{\mathrm{QS},\mathrm{exp}}$ appeared then to increase as collagen is gradually recruited at higher pressures, being, on average, 1.26 $\pm$ 0.08 and 1.58 $\pm$ 0.22 in the medium (120–80 mmHg) and high (160–120 mmHg) pressure range, respectively. The modelled stiffness ratios followed a similar increasing trend with pressure: 1.07 $\pm$ 0.06, 1.32 $\pm$ 0.11 and 1.54 $\pm$ 0.15 in the low-, medium-, and high-pressure range, respectively, at 10 Hz (Fig. 5B). While modelled stiffening ratios were, on average, higher than experimental values at low and mid pressures, these differences were attributable to samples IV and V, whose quasi-static highly nonlinear, biaxial mechanical behaviours were more difficult to capture using the proposed model (Table 1). This observation also explains the variability of ${k}_{2}^{2}$ and ${k}_{2}^{\mathrm{3,4}}$ parameters between different arteries (Table 1).

**Table 1:** Constituent-based quasi-linear viscoelastic model parameters of the *n* = 5 mouse carotid arteries tested in this study

Sample	Elastin					Collagen								Fit error	
	$\mu$ [kPa]	${\lambda }_{\theta ,\mathrm{e}}$ [−]	${\lambda }_{z,\mathrm{e}}$ [−]	${\nu }_{\mathrm{e}}$ [−]	${\tau }_{2,\mathrm{e}}$ [s]	${k}_{1}^{\mathrm{1,2},\mathrm{3,4}}$ [kPa]	${k}_{2}^{1}$ [−]	${k}_{2}^{2}$ [−]	${k}_{2}^{\mathrm{3,4}}$ [−]	${\alpha }^{\mathrm{3,4}}$ [°]	${\lambda }_{\mathrm{c}}$ [−]	${\nu }_{\mathrm{c}}$ [−]	${\tau }_{2,\mathrm{c}} \, [\mathrm{s}]$	RMSE_D_ [−]	RMSE_QS_ [−]
I	27.7	1.79	1.92	0.000	−	56.3	4.3	27.5	72.9	42.4	1.08	0.059	59.3	0.049	0.044
II	46.8	1.56	1.62	0.033	100.0	7.1	3.9	14.6	25.1	44.3	1.17	0.061	46.2	0.035	0.059
III	30.0	2.01	2.05	0.000	–	137.5	3.7	16.1	49.1	42.1	1.07	0.104	76.4	0.069	0.060
IV	21.7	1.84	1.83	0.000	–	62.2	0.0	3.9	8.8	42.4	1.19	0.137	20.6	0.076	0.092
V	15.2	2.07	1.92	0.000	–	86.4	3.0	3.0	19.7	44.5	1.12	0.135	17.7	0.090	0.078

Note that when ${\nu }_{\mathrm{e}}=0$, elastin behaves as a purely elastic material and a value for ${\tau }_{2,\mathrm{e}}$ cannot be estimated (i.e. any value of ${\tau }_{2,\mathrm{e}}$ yields the same modelled behaviour)

RSME_D_: root mean square error of the dynamic fitting (step 2 in Fig. 2); RSME_QS_: root mean square error of the quasi-static fitting (step 3 in Fig. 1). Note that ${\lambda }_{z,\mathrm{e}}$ was fixed to the ratio between the *in vivo* and unloaded length of the tested artery

As for the stiffness ratio, the loss factor showed considerable pressure dependency, being much lower in the low (0.021 $\pm$ 0.013) than in the medium (0.072 $\pm$ 0.011) and high (0.057 $\pm$ 0.006) pressure ranges (Fig. 5D). Once more, the increasing trend with pressure indicates that viscous effects are stronger in collagen-dominated parts of the pressure-diameter relationship. The modelled loss factor followed closely this trend, being 0.030 $\pm$ 0.011, 0.074 $\pm$ 0.016, and 0.080 $\pm$ 0.017 at 80–40, 120–80, and 160–120 mmHg, respectively (Fig. 5E).

### Comparison of constituent-based and standard quasi-linear viscoelastic models

**Fig. 6:**
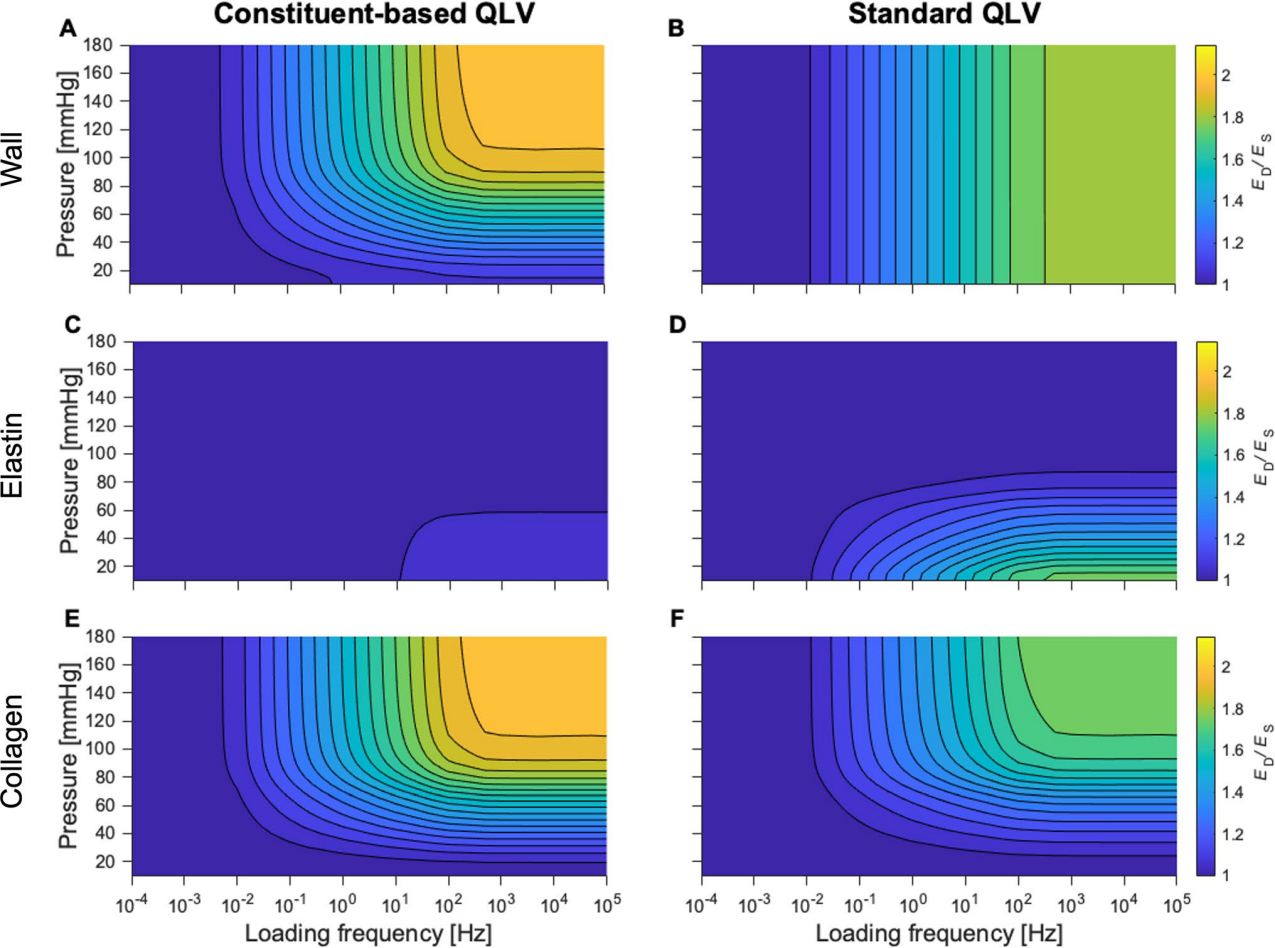
Average dynamic-to-static stiffness ratios as a function of pressure and loading frequency for the five mouse carotid arteries tested in this study show the difference between standard and constituent-based quasi-linear viscoelastic modelling. Panel **A**: constituent-based quasi-linear viscoelastic model. Panel **B**: standard quasi-linear viscoelastic model. Panels **C** and **D** show the contribution of elastin to the wall viscoelastic behaviour for the constituent-based and standard quasi-linear viscoelastic model, respectively. Panels **E** and **F** show the contribution of collagen to the wall viscoelastic behaviour for the constituent-based and standard quasi-linear viscoelastic model, respectively. Unlike in Fig. 5, here, the static and dynamic stiffnesses are calculated from the 2nd Piola-Kirchhoff stress–Green-Lagrange strain curves to show the effect of the quasi-linear viscoelasticity assumption. Furthermore, the amplitude of sinusoidal pressure waveforms was set to 0.5 mmHg to minimise the effect of the elastic nonlinearity on the calculated ratios. As inter-subject variability could not be effectively visualised in these graphs, individual plots are reported in the Supplemental Material, Figures S3 and S4

Model parameters for the sQLV model are presented in Table 2. Imposing ${\nu }_{\mathrm{e}}={\nu }_{\mathrm{c}}$ and ${\tau }_{2,\mathrm{e}}={\tau }_{1,\mathrm{e}}$ (i.e. using sQLV) affected the overall quality of the fitting of the experimental data, particularly increasing the dynamic fitting RMSE to 0.088 $\pm$ 0.033 (paired samples Student’s t-test, *p* = 0.035 versus cbQLV). The viscous coefficient ${\nu }_{\mathrm{e}}={\nu }_{\mathrm{c}}$ was 0.082 $\pm$ 0.030 and the time constant ${\tau }_{2,\mathrm{e}}={\tau }_{2,\mathrm{c}}$ was 29.6 $\pm$ 14.4 s, leading to a stress relaxation at $t\to +\infty$ of 44 $\pm$ 8% for both elastin- and fibre-borne parts of the wall stress. Note that this value is between the values of ${\nu }_{\mathrm{e}}$ and ${\nu }_{\mathrm{c}}$ as estimated in cbQLV. The ability of the sQLV model to capture the pressure dependency of ${\mathcal{K}}_{\mathrm{D}}/{\mathcal{K}}_{\mathrm{QS}}$ was considerably reduced compared to the cbQLV model: ${\mathcal{K}}_{\mathrm{D},\mathrm{mod}}/{\mathcal{K}}_{\mathrm{QS},\mathrm{mod}}$ at 10 Hz was 1.22 $\pm$ 0.17 at 80–40 mmHg, 1.40 $\pm$ 0.12 at 120–80 mmHg, and 1.47 $\pm$ 0.15 at 160–120 mmHg (Fig. 5C). The residual pressure dependency of ${\mathcal{K}}_{\mathrm{D},\mathrm{mod}}/{\mathcal{K}}_{\mathrm{QS},\mathrm{mod}}$ in sQLV is due to two factors: first, the stiffness ratio shown in Fig. 5 was evaluated in the current configuration; second, the applied pressure pulses spanned over 40 mmHg. Indeed, when these two effects are removed (i.e. computing ${E}_{\mathrm{D}}/{E}_{\mathrm{S}}$ in the reference configuration and using pressure pulses of negligible amplitude; Fig. 6), the difference between sQLV and cbQLV is more pronounced. In sQLV, ${E}_{\mathrm{D}}/{E}_{\mathrm{S}}$ is a function of the loading frequency alone, displaying a pressure-independent linear relationship with the logarithm of the loading frequency in the range $1/{\tau }_{2}$–$1/{\tau }_{1}$ (Fig. 6B). Conversely, in cbQLV, ${E}_{\mathrm{D}}/{E}_{\mathrm{S}}$ becomes a function of both loading frequency and pressure (or initial deformation), reflecting the constituent-specific viscoelastic properties and relative contributions to the total wall stress (Fig. 6A). The constituent-specific contributions to the wall viscoelasticity are presented in Fig. 6C and E for the cbQLV model and Fig. 6D and F for the sQLV model.

**Table 2:** Standard quasi-linear viscoelastic model parameters of the *n* = 5 mouse carotid arteries tested in this study

Sample	Elastin			Collagen						Viscous		Fit error	
	$\mu$ [kPa]	${\lambda }_{\theta ,\mathrm{e}}$ [-]	${\lambda }_{z,\mathrm{e}}$ [-]	${k}_{1}^{\mathrm{1,2},\mathrm{3,4}}$ [kPa]	${k}_{2}^{1}$ [-]	${k}_{2}^{2}$ [-]	${k}_{2}^{\mathrm{3,4}}$ [-]	${\alpha }^{\mathrm{3,4}}$ [°]	${\lambda }_{\mathrm{c}}$ [-]	${\nu }_{\mathrm{e}}={\nu }_{\mathrm{c}}$ [-]	${{\tau }_{2,\mathrm{e}}=\tau }_{2,\mathrm{c}} \, [\mathrm{s}]$	RMSE_D_ [-]	RMSE_QS_ [-]
I	40.7	1.79	1.92	50.9	4.5	27.6	73.5	42.4	1.08	0.046	38.6	0.062	0.049
II	53.7	1.56	1.62	6.8	3.9	14.6	25.1	44.3	1.17	0.054	50.9	0.037	0.059
III	52.9	2.01	2.05	118.9	4.4	16.6	50.7	42.4	1.08	0.079	30.8	0.114	0.076
IV	43.4	1.84	1.83	59.2	0.0	3.8	8.8	42.4	1.19	0.117	11.7	0.101	0.095
V	31.0	2.07	1.92	79.9	3.3	3.1	19.9	44.6	1.12	0.115	16.2	0.125	0.091

RSME_D_: root mean square error of the dynamic fitting (step 2 in Fig. 2); RSME_QS_: root mean square error of the quasi-static fitting (step 3 in Fig. 1). Note that ${\lambda }_{z,\mathrm{e}}$ was fixed to the ratio between the *in vivo* and unloaded length of the tested artery

As for the stiffness ratio, the loss factor in the sQLV model showed lower pressure dependency than that observed experimentally, being 0.055 $\pm$ 0.030, 0.075 $\pm$ 0.022 and 0.075 $\pm$ 0.019 vs 0.021 $\pm$ 0.013, 0.072 $\pm$ 0.011 and 0.057 $\pm$ 0.006 at 80–40, 120–80 and 160–120 mmHg, respectively (Fig. 5D and F).

## Discussion

The aim of the present study was to develop a viscoelastic constitutive model that captures the nonlinear viscoelastic response of the arterial wall using a unique set of deformation-independent model parameters. This was achieved by applying Fung’s quasi-linear viscoelastic (QLV) formulation at the *constituent* level rather than at the *whole-tissue* level. The proposed constituent-based QLV (cbQLV) approach captured the nonlinear viscoelastic behaviour of the mouse common carotid artery well over a wide range of quasi-static and dynamic deformations (see Supplemental information Figure S6) and performed better than standard QLV (sQLV). Furthermore, unlike sQLV, cbQLV allowed to identify the preponderant role of the fibrous constituents of the arterial wall (i.e. mainly collagen) in determining the viscous properties of the arterial wall.

In agreement with previous studies (Craiem et al. 2008; Zou and Zhang 2011; Yang et al. 2011; Amabili et al. 2019a; van der Bruggen et al. 2021a; Franchini et al. 2021), we found the mouse carotid artery to exhibit a fully nonlinear viscoelastic behaviour. The dynamic-to-quasi-static stiffness ratio calculated in the current configuration (i.e. in terms of Cauchy stress and engineering strain) increased by more than 1.5-fold from the low (80–40 mmHg) to the high pressure range (160–120 mmHg). These results are in agreement with those found in our previous study, although there observed in terms of structural and not material stiffness (van der Bruggen et al. 2021a). Other groups have reported similar increasing trends of arterial wall viscosity with increasing deformation/load. Franchini et al. (2021) subjected circumferential and axial strips of human aortic tissue to quasi-static uniaxial extension and harmonic loading at three different levels of initial stress. Similar to our experimental protocol, these initial stress levels corresponded to low, physiological, and high pressures. They found that the circumferential dynamic-to-quasi-static stiffness ratio defined in the reference configuration (i.e. in terms of 2nd PK stress and Green–Lagrange strain) increased by 1.25-fold from low to high initial stress. Similar findings have been reported for stress-relaxation experiments. Yang et al. (2011), Zou and Zhang (2011), and Peña et al. (2011) subjected strips of porcine aortas to uniaxial stress-relaxation tests in both circumferential and axial directions. They found that, for both tested directions, the degree of stress relaxation increased with the applied stretch (~ 1.5 to 2.5-fold).

Our cbQLV viscoelastic model showed good ability to capture the measured nonlinear viscous behaviour. The modelled gradient in stiffness ratio was close to that observed experimentally and much higher than the ~ 1.20-fold increase captured by sQLV. The cbQLV model attributed the carotid wall viscous behaviour predominantly to the fibrous part of our model, which is thought to mainly reflect the mechanical response of collagen fibres at high pressures. Conversely, elastin behaved as a purely elastic material in four of the five tested arteries. This result is not surprising considering how the load bearing of elastic arteries gradually shifts from elastin to collagen as pressure increases from sub-/low-physiological to supra-/high-physiological levels (Wolinsky and Glagov 1964; Berry et al. 1975; Sokolis et al. 2006). By attributing negligible viscoelastic behaviour to elastin and considerable stress-relaxation to the fibre-borne part of the stress, the model was able to simultaneously guarantee negligible dynamic stiffening at low pressures and more than 50% stiffening above physiological pressures. Indeed, these findings are in agreement with previous experimental observations on the effect of enzymatic digestion of collagen fibres on arterial wall mechanics (Zou and Zhang 2011; Weisbecker et al. 2013; Schriefl et al. 2015). It has been reported that softening behaviour (i.e. changes in the shape of the stress–strain relationship with increasing number of loading/unloading cycles) of human aortic wall samples subjected to cyclic uniaxial tensile testing is almost entirely attributable to collagen, and that collagen-digested medial samples exhibited a nearly linearly elastic behaviour with negligible softening (Weisbecker et al. 2013; Schriefl et al. 2015). This finding is also consistent with the previously reported increase of the stiffness ratio of the human aorta with age (Amabili et al. 2020). Similarly, Zou and Zhang (2011) tested the stress-relaxation of (1) intact, (2) decellularized, and (3) collagen-digested porcine aortic samples. They showed a progressive decrease of stress relaxation after removal of vascular smooth muscle cells (VSMCs) and collagen fibres. Furthermore, they found that the deformation-dependency of the stress-relaxation of the isolated aortic elastin matrix was considerably reduced compared to that of the intact tissue, which supports the validity of our cbQLV approach. These experimental findings suggest that, although cbQLV is likely still a simplification of reality (e.g. it neglects the passive contribution of VSMCs to the wall viscoelasticity), it may be able to effectively capture the different roles of elastin and collagen in the arterial wall viscoelastic behaviour.

Computational constitutive models are not only useful tools to provide a plausible micro-structurally motivated interpretation of complex, nonlinear, biaxial mechanical data; they also allow the estimation of an artery’s response to any prescribed loading condition. In the field of arterial viscoelasticity, previous fully nonlinear viscoelastic models have suffered from the necessity of defining deformation-dependent viscous parameters that would allow to capture the fully nonlinear (i.e. nonlinearity in both elastic and viscous behaviour), anisotropic behaviour of the arterial wall. This issue is often resolved through the definition of different sets of model parameters for each tested loading condition, yielding a model incapable of describing the wall behaviour over a wide range of loading conditions (Provenzano et al. 2002; Yang et al. 2011; Franchini et al. 2021). The main advantage of our cbQLV approach, thus, is to capture fully nonlinear viscoelastic behaviours with a unique set of deformation-independent model parameters. This is achieved by defining constituent-specific quasi-linear viscoelastic models which *individually* exhibit deformation-independent viscosity. However, because of the constituent’s relative contributions to the arterial wall behaviour are deformation-dependent, their combined contributions yield deformation-dependent viscoelasticity at the *whole-wall* level. A second advantage of cbQLV is that it is, in principle, independent from the knowledge of the purely static behaviour of the tested tissue (i.e. when viscous phenomena are null), which is hard to assess experimentally. In the present study, we used the proposed cbQLV modelling approach to capture the vessel responses to both quasi-static and dynamic loading. Both experimental conditions were treated as ‘dynamic’ experiments (i.e. with the entire deformation history affecting the measured stress/deformation at any time point). The latter aspect is particularly important when considering that our quasi-static measurements, conducted at loading rates that are more than two orders of magnitude below those experienced by arteries *in vivo*, showed considerable hysteresis. This suggests that viscoelastic phenomena should not be neglected in the quasi-static experimental protocols typically used in the field (Franchini et al. 2021), and that neither quasi-static loading nor unloading responses can be used to approximate the static behaviour. It follows that basing the estimation of the viscous model parameters solely on the dynamic-to-quasi-static stiffness ratio as an approximation of the dynamic-to-static stiffness ratio may lead to inaccurate interpretations of the viscoelastic behaviour of the arterial tissue (Franchini et al. 2021).

The investigation of the complex viscoelastic behaviour of arteries requires (1) sophisticated biaxial testing set-ups and (2) an effective viscoelastic constitutive modelling framework to integrate the acquired mechanical data. In a previous work, our group developed a state-of-the-art biaxial testing set-up that allows subjecting murine arteries to pseudo-physiological dynamic loading conditions (van der Bruggen et al. 2021a). In the present study, we propose an effective and convenient modelling tool to relate the observed macroscopic (passive) arterial behaviours to the mechanical properties of the arterial wall’s main structural constituents. In future studies, the combination of such experimental and modelling tools will allow the investigation of alterations in viscoelastic mechanical properties associated with cardiovascular system-related diseases, particularly those altering the microstructure and, consequently, mechanics of collagen and elastin (e.g. diabetes and hypertension) (Spronck et al. 2021; van der Bruggen et al. 2021b).

### Limitations

The experimental set-up used in this study was developed to assess the viscoelastic behaviour of arteries in pseudo-physiological loading conditions with high accuracy. Nevertheless, as acknowledged in the Methods, some sub-sampling resolution time delays between the acquired waveforms may exist. To address this, we used the dynamic protocol steps at the lowest loading frequency (2.5 Hz) as reference to perform ad hoc alignment of the recorded signals for the other protocol steps, under the assumption that the loss factor of soft biological tissues is nearly independent from the loading frequency (Fung 1997; Franchini et al. 2021). As shown in the Supplementary information Figure S5, however, the pressure dependence of dynamic-to-quasi-static stiffness ratio was comparable before and after the applied correction of the time delays, so that this limitation is not expected to significantly alter the results of our study.

In cbQLV, a fully nonlinear viscoelastic behaviour is achieved by superimposing the contribution of a finite number of sQLV models, each representing the response of a single wall constituent. The choice of the number and behaviour of the constituents inherently depends on the modelled tissue (Vena et al. 2006; Thomas et al. 2009). In this study, we assumed the passive mechanical behaviour of the mouse carotid artery wall to be determined by the summed contribution of an isotropic matrix (representing elastin) and four families of fibres which mainly reflect the behaviour of collagen. We, therefore, neglected the passive contribution of smooth muscle cells. This choice was made to limit the number of model parameters, as not to incur overfitting. Indeed, the passive mechanical response of smooth muscle has been previously modelled using the same SEF used herein for collagen (Spronck et al. 2021), thus making the identification of a unique set of model parameters impossible unless microstructural information from microscopy data is used to constrain the parameter space. Nonetheless, previous studies have shown that smooth muscle plays an important role in the arterial wall viscoelastic response, even under passive conditions and that both decellurization and collagen digestion significantly affect the viscoelasticity of the aortic wall (Apter et al. 1966; Zou and Zhang 2011; Franchini et al. 2022a). Therefore, smooth muscle cells are likely partly responsible for the viscous behaviour attributed here to collagen. Furthermore, in this study, the arterial wall was modelled as a thin homogeneous membrane, neglecting its tri-layered structure, inter-layer differences in viscoelastic behaviours, and the transmural stress distribution (Amabili et al. 2019a; Franchini et al. 2021). Given that the relative contribution of the arterial layers to the wall mechanical response is pressure-dependent (Giudici et al. 2021a; Giudici and Spronck 2022), a more complex multi-layered cbQLV model with layer-specific parameters could further improve the description of the nonlinear viscoelasticity of arteries. Furthermore, because of the single-layer thin-wall assumption, treatment of residual stresses is greatly simplified in our modelling framework; because of the inclusion of deposition stretches in the model, each constituent may not be stress-free in ${\kappa }_{\mathrm{u}}$, but the transmural distribution of residual stresses cannot be captured (Giudici and Spronck 2022; Zhang et al. 2022).

The mechanical behaviour of the mouse carotid artery is highly nonlinear and anisotropic. To capture this behaviour, our model involved a relatively high number of unconstrained model parameters (*n* = 14) which were estimated over three consecutive optimisation steps. Because of this strongly nonlinear behaviour, the estimated parameters may not accurately capture the true mechanics of the arterial wall for loading conditions that differ considerably from those applied experimentally. However, our experimental protocol was specifically designed to yield a thorough viscoelastic characterisation of the artery over wide ranges of biaxial deformations which also include pseudo-physiological loading scenarios (Supplemental information, Figure S6). Within these boundaries, our model can be expected to capture well the viscoelastic behaviour of the tested arteries.

Finally, in cbQLV, nonlinearity in viscous behaviour is provided by the fact that the relative load bearing of the material constituents is deformation-dependent. This implies that, in agreement with experimental findings of previous works (Peña et al. 2011; Zou and Zhang 2011; Yang et al. 2011; Amabili et al. 2019a; Franchini et al. 2021), the modelled viscoelastic behaviour depends on both the acting pressure and the axial stretch. It is worth noting, however, that in our study the effect of axial stretch was only assessed in quasi-static protocol steps, thus providing limited information on its contribution to arterial viscosity. In future studies, the model accuracy could be further improved by subjecting the arterial wall to harmonic loading in the axial direction and/or to harmonic loading in the circumferential direction at different levels of constant axial stretch.

## Conclusion

In this study, we proposed a constituent-based quasi-linear viscoelastic modelling framework for vascular applications. Compared to other viscoelastic modelling approaches, this framework (1) shows improved capture of fully nonlinear viscoelastic behaviours while using a unique set of deformation-independent model parameters, and (2) provides insight into the individual contributions of wall constituents to the wall viscoelasticity. Such model performance favours practical application and will, therefore, advance the characterisation of arterial mechanics under the varying loading conditions of *in vivo* physiology.

### Supplementary Information

Below is the link to the electronic supplementary material.

Supplementary file1 (PDF 580 KB)
