# Supplementary material for: Constituent-based quasi-linear viscoelasticity: a revised quasi-linear modelling framework to capture nonlinear viscoelasticity in arteries
Source: Biomech Model Mechanobiol. 2023 May 2;22(5):1607–23. doi: 10.1007/s10237-023-01711-8 (PMC10511394; doi:10.1007/s10237-023-01711-8)
Supplement: Supplementary file 1 — Supplementary file1 (PDF 580 KB) [file 10237_2023_1711_MOESM1_ESM.pdf]

Constituent-based quasi-linear viscoelasticity: A revised quasi-linear modelling framework to capture non-linear viscoelasticity in arteries

Alessandro Giudici, Koen W.F. van der Laan, Myrthe M. van der Bruggen, Shaiv Parikh, Tammo Delhaas, Koen D. Reesink, Bart Spronck

Supplemental information

Preconditioning protocol

After mounting the carotid artery on the pipettes of the biaxial experimental set-up, the preconditioning protocol begins with the experimental estimation of the *in vivo* axial length of the vessel, which is defined as the length for which the axial force is nearly independent of the intraluminal pressure in the range 10–180 mmHg. The vessel is then stretched to 105% of the estimated *in vivo* length and two quasi-static pressure sweeps are performed (range 10–180 mmHg). This step is repeated for the axial lengths corresponding to 95% of the *in vivo* length and to the *in vivo* length. These six quasi-static pressure sweeps are then followed by 10 quasi-static axial force sweeps as follows. Two quasi-static axial force sweeps between 0 N and the maximum axial force reached in the quasi-static pressure sweeps are performed for each of these constant intraluminal pressures: 10, 60, 100, 140, 180 mmHg. As the end of the preconditioning, a new estimation of the *in vivo* axial length is performed to be used in the testing protocol.

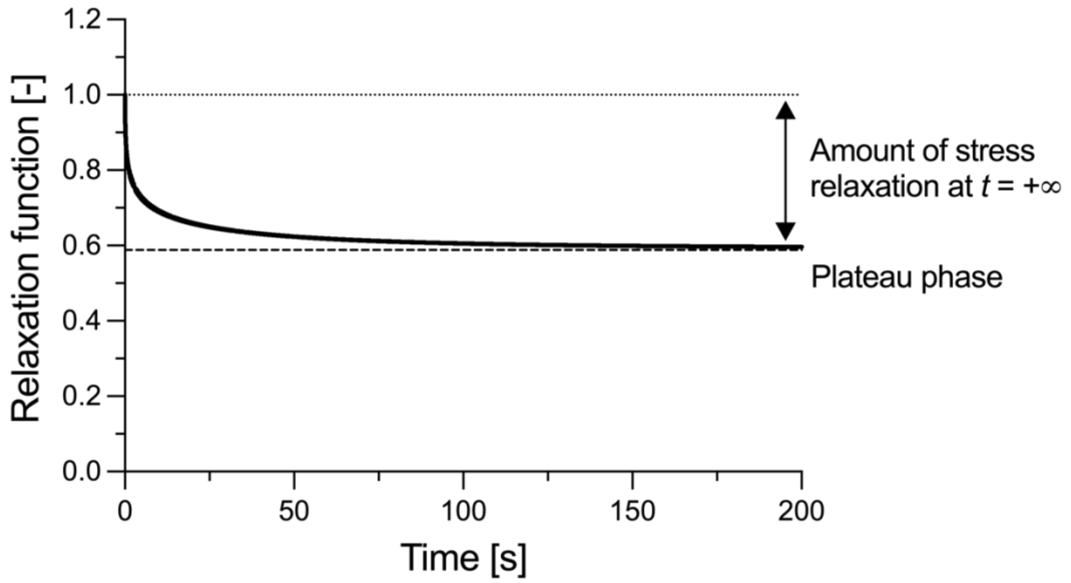

Figure S1 – Graphical representation of Fung's relaxation function (Eq.10) used in this study for $\nu = 0.09$, $\tau_1 = 0.05$ s, and $\tau_2 = 100$ s. The end relaxation asymptotic value (i.e., for $t \to +\infty$) can be analytically calculated from Eq.10 as $G(+\infty) = 1/\left[1 + \nu \ln\left(\frac{\tau_2}{\tau_1}\right)\right]$, so that the percent of stress relaxation for $t \to +\infty$ can be calculated as $[1 - G(+\infty)] \cdot 100\%$.

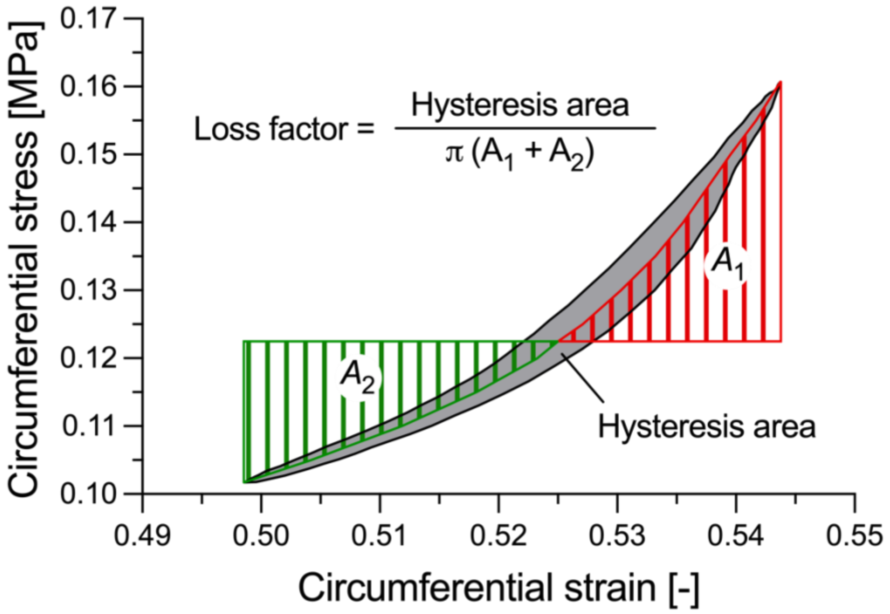

Figure S2 – Graphical representation of the calculation of the loss factor as the ratio.

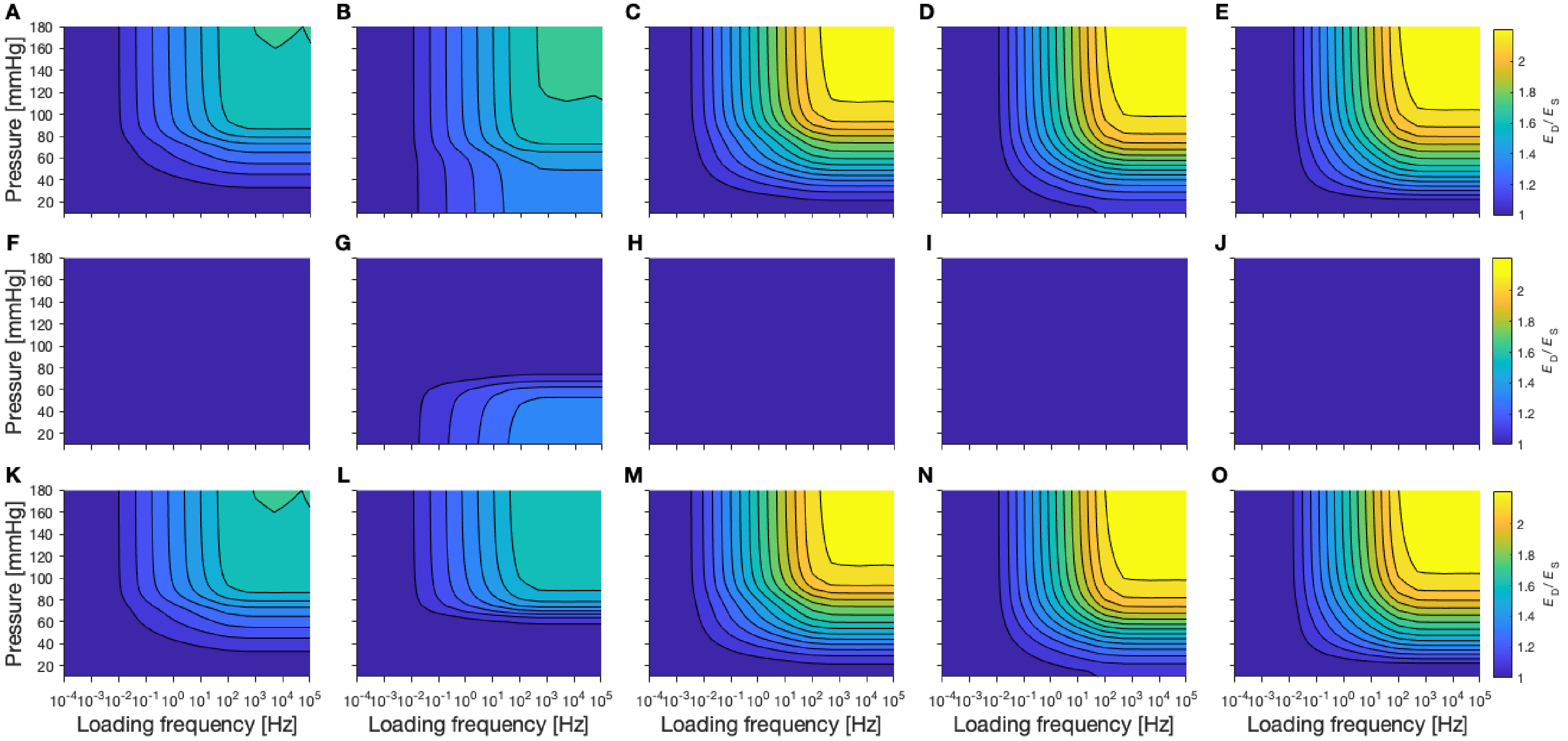

Figure S3 – Panel **A–E:** Dynamic to static stiffness ratio as a function of pressure and loading frequency for the mouse carotids I–V, respectively, as predicted by the constituent-based quasi-linear viscoelastic model. Panel **F–J**: Contribution of elastin to the wall viscoelastic behaviour for carotids I–V, respectively. Panel **K–O**: Contribution of collagen to the wall viscoelastic behaviour for carotids I–V, respectively.

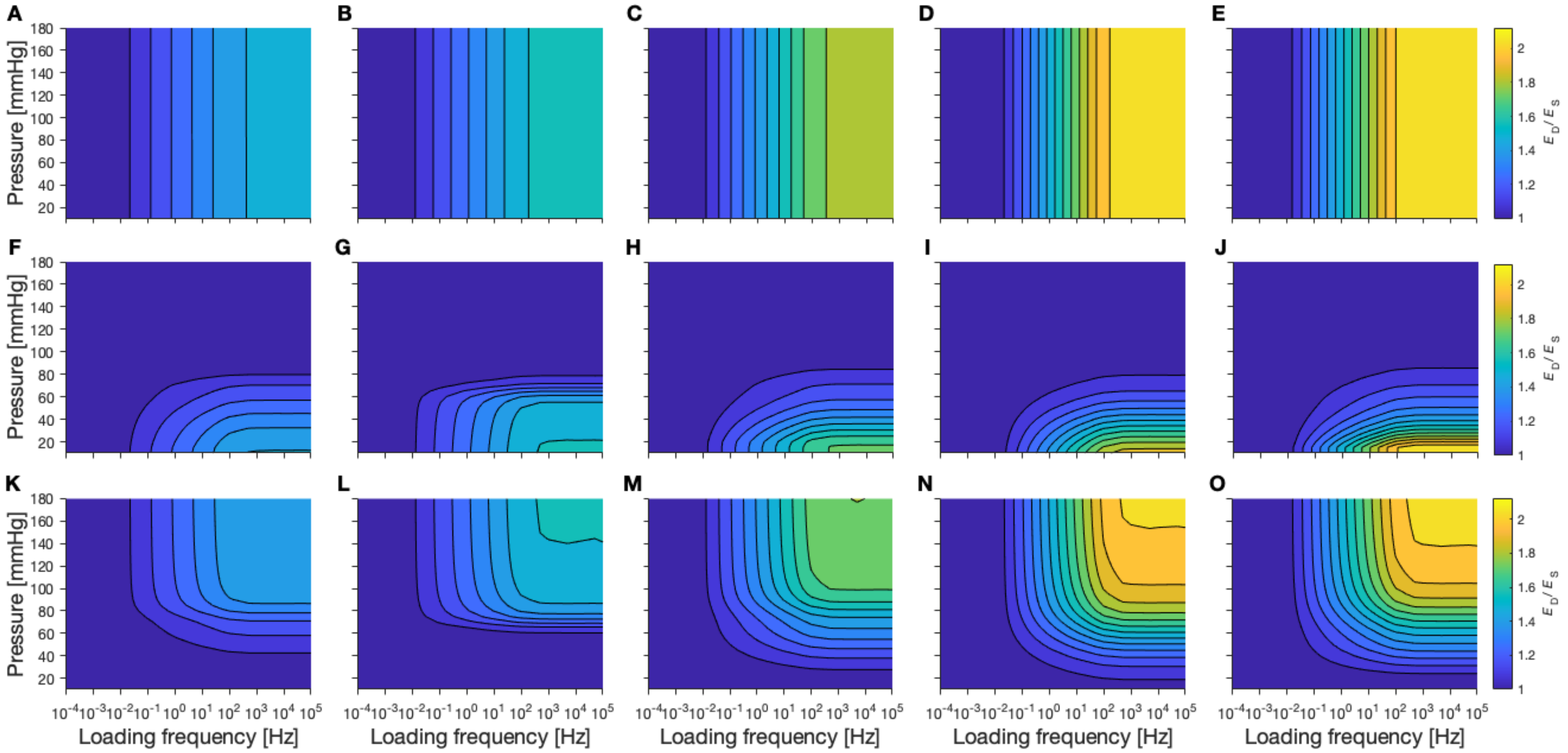

Figure S4 – Panel **A–E:** Dynamic to static stiffness ratio as a function of pressure and loading frequency for the mouse carotids I–V, respectively, as predicted by the standard quasi-linear viscoelastic model. Panel **F–J**: Contribution of elastin to the wall viscoelastic behaviour for carotids I–V, respectively. Panel **K–O**: Contribution of collagen to the wall viscoelastic behaviour for carotids I–V, respectively.

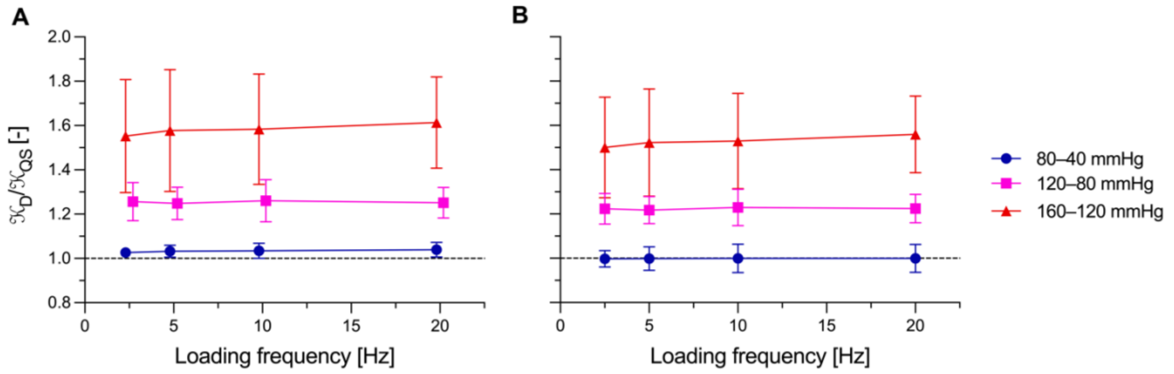

Figure S5 – Experimental dynamic-to-quasi-static stiffness ratio calculated after (Panel **A**) and before (Panel **B**) the pressure-diameter synchronisation adjustments described in Section 3.3.1. As visible, this ad-hoc signal alignment did not affect the pressure-dependency of the dynamic-to-quasi-static stiffness ratio.

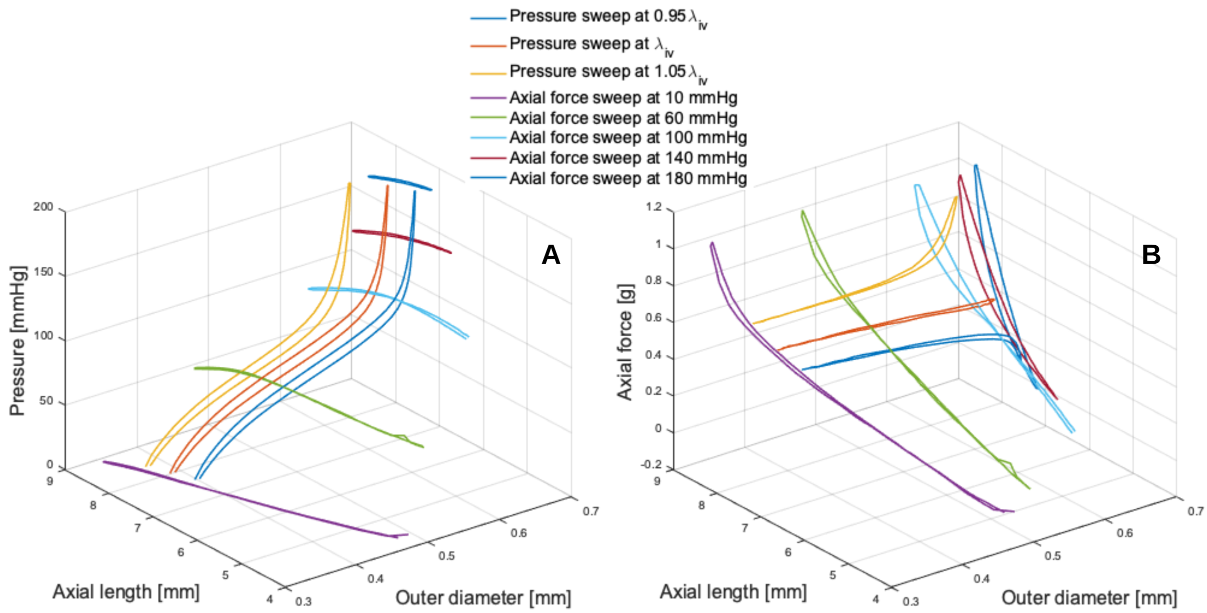

Figure S6 – Three-dimensional graph illustrating the deformation space explored through the quasi-static experimental protocol used in this study. Panel A shows the intraluminal pressure plotted as a function of the vessel axial length and outer diameter for the eight quasi-static experimental steps included in our protocol. Panel B shows the axial force plotted as a function of the vessel axial length and outer diameter for the same experimental steps. The proposed protocol provides a thorough characterisation of the artery mechanical behaviour in pseudo-physiological loading conditions. This enhances the physiological relevance of the proposed constituent-based quasi-linear viscoelastic model.
